# Supplementary material for: Why people follow rules
Source: Nat Hum Behav. 2025 May 26;9(7):1342–54. doi: 10.1038/s41562-025-02196-4 (PMC12283409; doi:10.1038/s41562-025-02196-4)
Supplement: Supplementary file 1 — Supplementary details on methods, Supplementary Figs. 1 and 2, Tables 1–11 and Materials: instructions shown to participants. [file 41562_2025_2196_MOESM1_ESM.pdf]

nature human behaviour

Supplementary information https://doi.org/10.1038/s41562-025-02196-4

Why people follow rules

In the format provided by the authors and unedited

Contents

1. **Supplementary Details on Methods** **p. 2**
 1.1. Procedures common across all experiments p. 2
 1.2. The experiments p. 2
 1.3. Replications of the baseline experiments p. 4
 1.4. Eliciting social expectations: Normative beliefs b^n p. 5
 1.5. Eliciting social expectations: Descriptive beliefs b^d p. 5
 1.6. Eliciting normative and descriptive conditional rule-conformity functions $n(b^n), d(b^d)$ p. 5
 1.7. Statistical analysis p. 6

2. **Supplementary Figures**
 Fig. S1. Absolute rule-following levels across 27 treatments of the traffic light task in the presence of peers (iteration 2) p. 7
 Fig. S2. Absolute rule-following levels in iteration 3 of the traffic light task after observing peers p. 7

3. **Supplementary Tables** **p. 8**
 Table S1. Overview of experiments and demographics of participants p. 8
 Table S2. Descriptive statistics of participants in the rule-following task p. 9
 Table S3. Determinants of rule-conformity when participants complete the task alone (iter. 1) p. 10
 Table S4. Rule-conformity is conditional on normative and descriptive beliefs p. 11
 Table S5. Rule-conformity in the presence of peers (iteration 2) p. 12
 Table S6. Rule-conformity in the presence of peers (modelling peer rule-conformity rates in iteration 2 as a continuum) p. 13
 Table S7. Rule-conformity *after* observing peers (iteration 3) p. 14
 Table S8. Peer behaviour shifts perceived social appropriateness p. 15
 Table S9. Effects of externalities and punishment on social expectations p. 16
 Table S10. Conditional rule-conformity functions in the presence of externalities and punishment p. 17
 Table S11. Determinants of rule-conformity in the presence of externalities and punishment p. 18

4. **Supplementary Materials: Experimental instructions shown to participants** **p. 19**
 4.1. Rule-following task (traffic light task) across all experiments p. 19
 4.2. Eliciting normative beliefs b^n p. 35
 4.3. Eliciting descriptive beliefs b^d p. 40
 4.4. Eliciting the normative conditional rule-conformity function $n(b^n)$ p. 46
 4.5. Eliciting the descriptive conditional rule-conformity function $d(b^d)$ p. 52
 4.6. Eliciting normative expectations before and after observing peer behaviour p. 57
 4.7. Abstract version of the rule-following task p. 66

5. **Supplementary References** **p. 68**

1. Details on Methods

1.1. Procedures common across all experiments

The experiments received ethical approval from the Research Ethics Committee of the School of Economics at the University of Nottingham (protocol id 030_ERC_AP_MT) and were conducted between 2014 and 2016 (data associated with Figs. 1-3; Extended Data Fig. 1) and in 2018 (Fig. 4). Participants provided informed consent before entering the experiments.

With one exception (see Section 1.3), participants were recruited from Amazon Mechanical Turk (AMT). AMT is a web-based work platform (https://www.mturk.com/) frequently used for scientific research[1,2]. Unless stated otherwise, participation was restricted to AMT workers from the USA to ensure understanding of English instructions (see Table S1 for demographic details of all experiments reported in this paper). The only other requirement we used was for workers to have an approval rating of at least 90% (thought to be sufficient at the time of the experiments (2014-18)) based on previous tasks they had completed on AMT.

Participants were recruited by posting 'Human Intelligence Tasks' (HITs) which they could accept and complete at will. The HIT descriptions always stated the estimated length of the task, the possible range of bonus payment, and the guaranteed participation completion fees ($0.50 in all experiments). To minimize assortative selection into our experiments, the HIT descriptions only contained general information on the task contents ("This is a study on decision making").

To start their task, participants clicked on a link directing them to the experimental pages, which they completed in their web browsers. Upon completion, participants received a unique code to collect their performance-specific payment via AMT. For further methodological discussions of using AMT for behavioural research, see, e.g., refs[1,2]. We used the software LIONESS Lab (https://lioness-lab.org/)[3] for developing and conducting our experiments. Note that all experiments were incentivized and at no point did we use deception.

Below we describe each of our experiments. Participants' on-screen instructions and decision screens are provided in the Section 'Experimental Materials'.

1.2. The experiments

The tasks

In most experiments we used a "traffic light task", which is a simplified and more abstract version of a related tool developed by Kimbrough and Vostroknutov (which had five traffic lights, a stick figure, and a "Walk" button)[4]. In our simplified and more abstract task, participants controlled a circle and had to "move" it across the screen to a single red traffic light, and then over a finish line (Fig. 1a of the main text). Participants started with an endowment of 20 money units (MU), which was worth $1.00. Each second this endowment was reduced with 1 MU, indicated at the top of the screen. Across all treatments, the traffic light turned green after 12 seconds. The instructions (see Section 4.1 of SI) made it clear that the participant would make the most money by moving the circle across the finish line as fast as possible; that is, before the traffic light turned green. However, following Kimbrough and Vostroknutov[4], the instructions also explicitly stated that "The rule is to wait until the traffic light turns green". It was clear that there was no sanction for violating this rule (except in treatments with punishment, see below).

The abstract version of the task was developed by us and modelled on our version of the traffic light task (see Fig. 1b): instead of the traffic light there was a X across a grey waiting area. The rule said: "The rule is to wait in the grey area until the cross disappears". The purpose of this task was to see whether an even more abstract task leads to a different rate of rule-following than the slightly more contextualised traffic light task. For the instructions see Section 4.7 of this SI.

Experimental conditions

In total, 7,732 participants completed the traffic light task, in two conditions: without and with control questions. In the condition with control questions (n = 4,970), after the instructions, but before the actual task, we included two compulsory control questions about possible earnings for rule violation and rule-following to maximize participants' understanding of how earnings were computed. In the condition without control questions (n = 2,762), no questions were asked. For the instructions to participants (including treatment variations), see Section 4.1 of this SI.

The abstract task was only run with control questions (instructions are in Section 4.7) and 1,251 AMT workers took part in it.

For treatments with *externalities* (EX, WP, SP, see Fig. 4 in the main text), the instructions stated that, in addition to their own earnings, participants could earn $1.00 for a well-known charity (the Red Cross) if they conformed with the rule. Violating the rule would reduce the earnings for the Red Cross to $0.00.

For treatments with *punishment* (Weak Punishment, WP; and Strong Punishment, SP), the instructions stated that the computer would inspect the participant's decision in the task with a certain probability (10% for the WP treatment, and 90% for the SP treatment). This inspection only had payoff consequences when a participant had violated the rule. In those cases, the participant's earnings were reduced to $0.00.

Each participant completed three iterations of the traffic light task, following an ABA design:

1. In the *first iteration*, referred to below as Part I, the participant completed the task alone (this data is the basis for Fig. 1c,d).
2. In the *second iteration* (called Part II), we implemented 27 treatments, systematically varying the number of peers a participant observed (from 1 to 6), and in how many of these peers followed or violated the rule (Fig. 3). In the 28th treatment (the baseline treatment, the same as Experiment 1), no peers were shown in B. So, for participants in that treatment, the task in B was identical to A.
3. The *third iteration* (called Part III) was the same as the first iteration. Participants again completed the task alone.

In each case, we measured the time at which a participant moved the circle across the screen. Moving before the traffic light turned green counted as rule-breaking and moving after the traffic light turned green counted as rule-following (see Fig. 1).

Payments

Participants took 5-7 minutes to complete the traffic light task. On top of their completion fee of $0.50, participants received a performance-specific bonus that depended on their decision in the task and the rules of the experiment. Average bonuses were $0.45 (range $0.00 to $0.85, with peaks at $0.35 for rule-following and $0.85 for rule-violations), making for an average participant hourly wage of $9.50. Further details are described in Sections 1.4 to 1.6.

1.3 Replications of the baseline experiment

We conducted most experiments online, but we also report a replication of our rule-following task in a traditional experimental economics laboratory which affords a higher degree of control than an online environment. The experiment with student participants we report in the main text (and in Extended Data Fig. 1) were conducted in 2016 with undergraduate volunteer participants (*n*=103; mean age: 21.0 (s.d.=2.0); 47% male) in the experimental economics research laboratory in the Centre for Decision Research and Experimental Economics (CeDEx) at the University of Nottingham, UK[1]. The setup was identical to the online traffic light task with control questions, except that 20 points equalled GBP 1 rather than USD 1 (as in the online experiments), aligning with average hourly payment in the CeDEx laboratory. As reported in the main text and in Extended Data Fig. 1, the rate of rule-following (in the traffic light task with control questions) is 60%, which is similar to the 58% rate of rule-following observed online. This suggest that the inevitable differences between a university research lab and an online lab do not matter for rule-conformity in our task (like in many experiments that tested for the difference between lab and online experiments[5,6]).

Our replication is well in line with rule-following rates in our experiments, including the 28 between-subjects treatments reported in Fig. 3b in the main text, results from related studies on separate research questions cited in the main text, as well as published results from related tasks with various samples cited in the main text: an experiment with the serial traffic light task reported 64% rule-following[4], and, across different societies between 44% and 61% rule-following in the ball-division task (a more abstract version of the traffic light task)[7].

For completeness and transparency, we report one exception to these consistent results of baseline rule-following. In 2021 we ran an experiment with the traffic light task and the abstract task, with and without control questions (total *n*=381). We used a fresh AMT requester account and used the same selection criteria as in 2014-2015 and 2018 (approval rating of at least 90%, location: US). This led to a surprisingly low rule-following rate of 25% across three iterations of the no-peer baseline condition. However, we have serious concerns about the internal validity of this experiment: 60% of participants used IP-addresses suspected to be from VPN farms (based on the first 6 digits of the IP-address being stored in our records). Moreover, in our exit questionnaire we asked: "Do you happen to have participated in a HIT similar to this one, in which you had to move your circle across the screen?" (added because on the new requester account we had no access to the 'uniqueTurker' code we previously used to exclude re-takers across sessions). 40% of participants indicated that they had experience with the task (versus <2% in Prolific samples). Participants who indicated they participated in the task before had substantially lower rule-following rates (15%) than 'naïve' participants (32%). Focusing only on naïve participants with a non-suspicious IP address, we are left with only 40 participants who completed the traffic light task. Overall, we conclude that the internal validity of the experiment was very likely seriously compromised.

Based on some previous research on various topics that compared lab and AMT and found that lab and AMT samples behaved similarly[1,2,8,9], we have no reason to believe that our data, which we collected between 2014 and 2018, are affected by data quality issues. However, as we learned through our problems with data quality mentioned above, things might have changed in recent years[10,11]. Relative to 2014-2018, sampling on AMT requires much sharper vetting than focussing on approval ratings of even 95% (e.g., by using expert vetting services[12]), adding attention checks[13], and/or considering other platforms[14,15].

[1] See https://www.nottingham.ac.uk/cedex/cedex-labs/index.aspx

1.4. Eliciting social expectations: normative beliefs (b^n)

We measured normative beliefs in the traffic light task (Fig. 2a of the main text, and Extended Data Figs. 3 and 4a) using the incentive-compatible methodology of Krupka and Weber[16]. For the instructions see Section 4.2 of this SI. Participants were put in the role of a spectator and were asked to rate the 'social appropriateness' of behaviours of a hypothetical decision-maker participating in the traffic light task. After reading the instructions that this spectator would receive (these were the same as the instructions given to participants in the traffic light task), spectators were asked to evaluate two possible behaviours: (*i*) following the rule and (*ii*) violating the rule. Ratings were given on the following scale (with numerical value in brackets): (1) 'very socially inappropriate', (2) 'somewhat socially inappropriate', (3) 'somewhat socially appropriate', and (4) 'very socially appropriate'.

To measure how normative expectations were influenced by observing peer behaviour (Extended Data Fig. 3), the spectators first rated the social appropriateness of following and breaking the rule in a situation where the decision-maker would make their decision alone. Subsequently, they rated the same possible behaviours when the decision-maker was in the presence of peers.

After a session was over, spectators were randomly paired, and for each pair we randomly selected one rated action (follow or violate) in one setting (with or without peers). If the ratings matched, both spectators in a pair received \$2.00. Otherwise, they received \$0.00. Thus, the spectators had an incentive to report their true normative beliefs. Spectators were informed of this incentive scheme before they reported their normative belief.

Spectators took approximately 7 minutes to complete the task. On top of their completion fee of \$0.50, spectators earned an average bonus of \$0.80, making for an average participant hourly wage of \$11.15.

1.5. Eliciting social expectations: descriptive beliefs (b^d)

Participants were put into a role of a spectator and had to estimate the rule-following rate among previous AMT workers who participated in the traffic light task (Fig. 2b and 4 of the main text; Extended Data Fig. 4b). First, spectators were shown the instructions that these previous workers had received. Second, after confirming they understood the previous workers' task, spectators entered their estimate of the percentage of workers who obeyed the rule. If their estimate was no more than 5 percentage points off the observed percentage of rule-following, participants received a bonus payment of \$1.00. Otherwise, they received \$0.00. Thus, the spectators had an incentive to report their true descriptive beliefs. Spectators were informed of this incentive scheme before they participated in the task. For the instructions see Section 4.3 of this SI.

Spectators took approximately 5 minutes to complete the task. On top of their completion fee of \$0.50, participants earned an average bonus of \$0.30, making for an average participant hourly wage of \$9.60.

1.6. Eliciting normative and descriptive conditional rule-conformity functions $n(b^n), d(b^d)$

Participants in these experiments had to decide whether to follow or to violate the rule for different, exogenously induced levels of normative and descriptive beliefs (Fig. 2c,d in the main text and Extended Data Figs. 4c,d). To elicit these conditional rule-conformity functions, we used an approach based on the strategy method we had developed[17] and used in previous research in public goods games[18]; subsequent research on rule-following has also used the strategy method to elicit conditional conformity functions[19-21]. After reading the instructions, we asked participants to choose whether they preferred either to conform with

the rule (wait until the traffic light turned green) or to break it (move before the traffic light turned green) across five difference scenarios. In each case, participants were informed of the exact payoffs associated with rule-following and violation of the rule. These payoffs were based on the actual payoffs from rule-following and violation in the behavioural task.

In the case of *conditional rule-conformity as a function of descriptive beliefs* $d(b^d)$, we elicited participants' preference for rule-following for scenarios that differed in the percentage of other AMT workers who would follow the rule. In scenario 1 we told participants to imagine that between 0 and 20% of other AMT workers would follow the rule and asked them whether they themselves preferred to violate or follow the rule in this situation. In the other four scenarios we elicited participants' preference for following the rule in case 21-40%, 41-60%, 61-80% or 81-100% of other AMT workers would conform with the rule. Thus, the elicitation measures participants' conditional preference for rule-following $d(b^d)$, in correspondence to five *exogenous* levels of descriptive beliefs (b^d) about others' rule-following.

The elicitation for *conditional rule-conformity as a function of normative beliefs* $n(b^n)$ was similar except that we described different scenarios in which 0-20%, 21-40%, 41-60%, 61-80% or 81-100% of other AMT workers would disapprove of rule violations.

We used a between-subject design: AMT workers participated in the elicitation of either $n(b^n)$ or $d(b^d)$.

The elicitation was incentive compatible. Participants were told truthfully that one of the five scenarios corresponded to the actual outcome of a study we had previously run on AMT. The instructions (see Sections 4.4 and 4.5 of the SI) stated that, at the end of the experiment, we would use their response in this scenario to calculate payments ($0.35 for obeying the rule and $0.85 for breaking the rule; based on the behavioural task). The relevant scenarios were identified as follows. For descriptive beliefs, we used the share of AMT workers who conformed with the rule in the behavioural task (Section 1.5 of SI). For normative beliefs, we used the share of AMT workers who found rule violations socially inappropriate in the normative beliefs task (Section 1.4 of SI).

Participants took approximately 5-7 minutes to complete the task. On top of their completion fee of $0.50, participants earned an average bonus of $0.50, making for an average participant hourly wage of $10.00.

1.7. Statistical analysis

Tables S2-S6 and S9-S11 below report linear probability models fitted to participants' binary decisions to follow (1) or violate (0) the rule. Linear models are typically robust to deviations from normality assumptions[22], and their results are easier to interpret than models using a logit or probit link function to deal with the binary nature of the dependent variable. For all models we ran robustness checks by also fitting logistic models. For consistency with the other analyses, the models reported in Tables S2-S6 only include the demographic controls ‘age’ and ‘gender’. As additional robustness checks we also fitted linear models including all control variables. We report two-tailed statistical tests. Unless stated otherwise, the results reported in the Tables below are robust to these variations in the model specification.

Statistical analyses were conducted in *R*, v.4.0.2[23]. Mixed models were fitted using the package *lme4*[24] and multiple comparisons (Tukey’s HSD tests) were conducted with the package *multcomp*[25]. Data and commented code (including all robustness checks) are available in the repository (see https://osf.io/7wz4f/).

2. Supplementary Figures

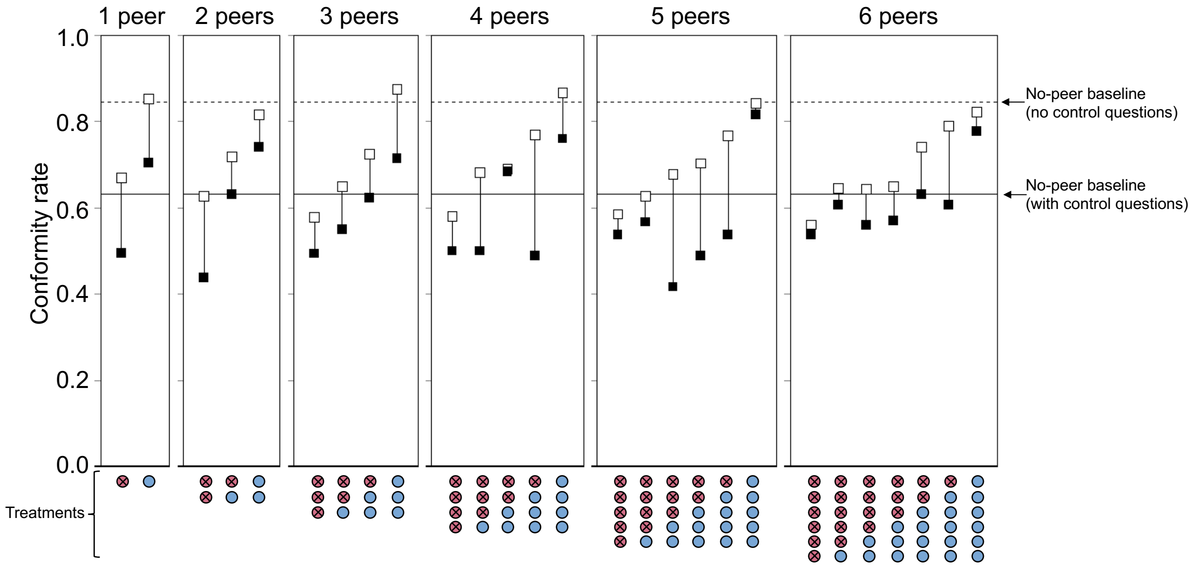

Fig. S1 | Absolute rule-following levels across 27 treatments of the traffic light task in the presence of peers (iteration 2). This shows raw rule-following rates underlying the data shown in Fig. 3c of the main text. Red circle with ×: Peer violated the rule; Blue circle: peer followed the rule. Solid and open square dots respectively show data with and without control questions. Solid and dashed horizontal lines respectively show no-peers baseline rule-following rates with and without control questions. Figure 3b in the main text shows average rule-following rates in the treatments and the no-peers baseline, weighted according to the number of data points underlying the treatment data. Note that in this data, which stems from the second iteration of the traffic light task, overall rule-following rates (including those in the no-peers baseline conditions) are higher than in the first iteration shown in Figs. 1c,d of the main text. For regression models fitted to these data, see Tables S4, S5.

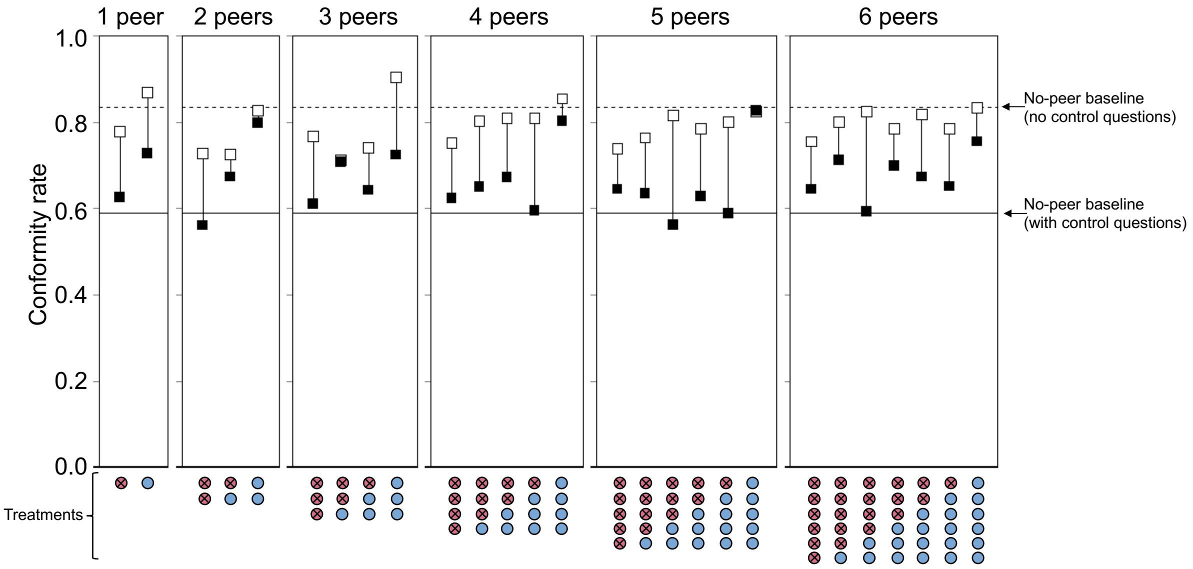

Fig. S2 | Absolute rule-following levels in iteration 3 of the traffic light task *after* observing peers. Same graph as Fig. S1, but now for the third iteration of the task, when participants completed it alone again but had experienced one of the 27 treatments. For regression models fitted to these data, see Table S6. Treatments: Red circle with ×: Peer violated the rule; Blue circle: peer followed the rule.

3. Supplementary Tables

Table S1 | Overview of experiments and demographics of participants. Demographic details (age and proportion male) are provided for each of the thirteen separate experiments run for this study.

Experiments	n	mean age (s.d.)	proportion male
**Experiments 1 and 3**			
Behavioural task (Figs. 1a,c,d and Fig. 3)	7,732	34.11 (11.26)	0.49
Abstract task (Fig. 1b,e)	1,251	34.89 (11.41)	0.55
Laboratory sample Nottingham (main text)	103	21.05 (1.96)	0.46
**Experiments 2**			
Normative beliefs (Fig. 2a)	99	37.22 (11.90)	0.41
Descriptive beliefs (Fig. 2b)	95	34.07 (9.03)	0.54
Conditional rule-conformity (normative; Fig. 2c)	159	31.76 (9.01)	0.50
Conditional rule-conformity (descriptive; Fig. 2d)	158	34.61 (10.37)	0.53
Normative beliefs (EDFig. 3)	392	37.25 (12.32)	0.49
**Experiments 4**			
Behavioural tasks (Fig. 4)	2,007	36.33 (11.47)	0.46
Externalities: normative beliefs (EDFig. 4a)	404	34.95 (11.22)	0.49
Externalities: normative beliefs (EDFig. 4b)	408	34.65 (10.39)	0.47
Externalities: cond. conformity (normative; EDFig. 4c)	607	35.11 (11.27)	0.46
Externalities: cond. conformity (descriptive; EDFig. 4d)	619	35.70 (10.94)	0.45
**Total**	**14,034**	**34.64 (11.31)**	**0.49**

Note: EDFig. denotes Extended Data Figure

Table S2 | Descriptive statistics of participants in the rule-following task (Fig. 1a,c,d). 7,732 people participated in these experiments. For binary variables we show proportions. Demographic variables include age and gender, which feature as controls in further analyses (*cf.* Tables S2-S10). For exploratory purposes, we measured several personality characteristics that we suspected might be associated with rule-following. 'Patience' refers to responses to the question "Are you generally an impatient person, or someone who always shows great patience?" (scale 0-10)[26]. 'Risk taking' refers to responses to the question "How do you see yourself: are you generally a person who is fully prepared to take risks or do you try to avoid taking risks?" (scale 0-10)[27]. 'Conditional cooperation' was measured with an unincentivized binary prisoner's dilemma game in which participants chose to either cooperate or defect for cases in which their partner would cooperate or defect (a binary version of a frequently-used paradigm to measure conditional cooperation[18]). Participants were characterised as a conditional cooperator (coded as 1) if they cooperated only if their partner did so as well (otherwise the variable was coded as a 0). Guilt and shame proneness are measured with the GASP scale[28]. The Big Five personality traits are measured with the 'Ten-Item Personality Inventory'[27]. 'Control questions' indicates the proportion of participants for whom control questions were present (1) or not (0).

Variable	Mean	St. Dev.	Min	Pctl(25)	Pctl(75)	Max
Age	34.1	11.3	18	26	40	80
Male (proportion)	0.490	0.500	0	0	1	1
Patience	6.278	2.551	0	4	8	10
Risk taking	5.557	2.359	0	4	7	10
Conditional cooperator (proportion)	0.617					
Guilt and shame proneness						
Guilt - negative behaviour-evaluation	5.418	1.300	1	4.75	6.5	7
Guilt - repair	5.664	0.997	1	5	6.5	7
Shame - negative self-evaluation	5.566	1.183	1	5	6.5	7
Shame - withdraw	2.991	1.195	1	2	3.8	7
Big Five personality traits						
Extraversion	3.538	1.663	1	2	5	7
Agreeableness	5.177	1.297	1	4.5	6	7
Conscientiousness	5.406	1.274	1	4.5	6.5	7
Emotional stability	4.767	1.553	1	3.5	6	7
Openness	5.101	1.290	1	4	6	7
Other						
Control questions (proportion)	0.357		0	0	1	1

Table S3 | Determinants of rule-conformity when participants complete the task alone (iteration 1). We show estimates of a linear probability model fitted to decisions to violate (0) or follow (1) the traffic light rule, with 95% confidence intervals in brackets. Violation and rule-conformity were defined as moving before and after the traffic light turned green, respectively (after 12 seconds; *cf.* Fig. 1b of the main text). Variables in this exploratory analysis are the same as in Table S2 and were elicited after the behavioural task for exploratory purposes. We observe that most effects are (very) small. One exception is the addition of control questions, which reduces rule-conformity. Rule-conformity tends to be slightly higher for participants who are younger, self-report as being more patient, and are prone to experience shame. Conditional cooperators tend to follow somewhat more often. 'Shame withdraw' is thought to be associated with antisocial behaviour[28]. Exploring correlations with the Big Five personality traits[27] reveals that agreeable, emotionally stable, and less open people are less likely to follow, but again, effects are very small. See Table S2 for references explaining the measurement of these items.

**Dependent variable**	**Rule conformity (0=no, 1=yes)**
Age	-0.002 (-0.003, -0.001), p<0.001
Male	-0.019 (-0.042, 0.004), p=0.100
Patience	0.033 (0.029, 0.037), p<0.001
Risk-taking	-0.002 (-0.007, 0.003), p=0.492
Conditional cooperator (0=no, 1=yes)	0.025 (0.003, 0.046), p=0.025
Guilt and shame proneness	
Guilt - negative self-evaluation	0.000 (-0.011, 0.011), p=0.993
Guilt - repair	0.010 (-0.003, 0.023), p=0.123
Shame - negative self-evaluation	0.015 (0.004, 0.026), p=0.010
Shame - withdraw	-0.025 (-0.034, -0.016), p<0.001
Big Five	
Extraversion	-0.004 (-0.011, 0.003), p=0.251
Agreeableness	-0.018 (-0.028, -0.009), p<0.001
Conscientiousness	-0.000 (-0.009, 0.010), p=0.961
Emotional stability	-0.025 (-0.033, -0.016), p<0.001
Openness	0.010 (0.001, 0.020), p=0.024
Other	
Control questions (0=no, 1=yes)	-0.119 (-0.140, -0.097), p<0.001
Constant	0.668 (0.566, 0.769), p<0.001
Observations	7,732
R^2	0.059
Adjusted R^2	0.057
Residual Std. Error	0.461 (df = 7716)
F Statistic	32.368 (df = 15; 7716), p<0.001

Table S4 | Rule rule-conformity is conditional on normative and descriptive beliefs. We show estimates of three linear probability models with 'participant' as random effect fitted to decisions to violate (0) or follow (1) the stated rule. 'Condition' reflects the % of people disapproving of a violation (for normative beliefs) or the % of people following the stated rule (for descriptive beliefs; *cf.* Figs. 2c,d in the main text). For both normative and descriptive beliefs, responses were elicited for quintiles 0-20, 21-40, 41-60, 61-80, 81-100 (see Methods, and Sections S1.3 and S1.4 in SI for details), and these quintiles enter the regressions as 10, 30, 50, 70 and 90, respectively. Models 1 and 2 only include data from the normative and descriptive beliefs treatments, respectively; model 3 was fitted to data from both treatments. The variable 'Treatment' refers to either normative (0) or descriptive (1) beliefs. We observe that for both normative and descriptive beliefs, slopes are strongly significantly positive, confirming the visual impression for Fig. 2c and 2d in the main text. This indicates strong conditionality for both types of social expectations. Conditionality on descriptive expectations is slightly stronger, as indicated by the significant interaction effect in Model 3.

	**(1) Normative beliefs**	**(2) Descriptive beliefs**	**(3) Both**
**Dependent variable**	**Conditional rule conformity (0=no, 1=yes)**		
Condition (% disapproving/following)	0.264<br>(0.175, 0.354)<br>p<0.001	0.389<br>(0.310, 0.469)<br>p<0.001	0.264<br>(0.180, 0.349)<br>p<0.001
Treatment (0=Normative, 1=Descriptive)			-0.123<br>(-0.226, -0.019)<br>p=0.020
Treatment × Condition			0.125<br>(0.005, 0.245)<br>p=0.041
Constant	0.249<br>(0.019, 0.480)<br>p=0.035	0.117<br>(-0.108, 0.342)<br>p=0.307	0.244<br>(0.082, 0.407)<br>p=0.004
Controls			
Age	0.004 (-0.003, 0.010)<br>p=0.238	0.003 (-0.003, 0.009)<br>p=0.316	0.003 (-0.001, 0.008)<br>p=0.116
Male	-0.081 (-0.196, 0.035)<br>p=0.171	-0.005 (-0.126, 0.115),<br>p=0.930	-0.044 (-0.127, 0.040)<br>p=0.305
Number of participants	159	158	317
Observations	795	790	1,585
Log Likelihood	-469.857	-395.073	-864.445
Akaike Inf. Crit.	951.714	802.145	1,744.890
Bayesian Inf. Crit.	979.784	830.178	1,787.837

Table S5 | Rule-conformity in the presence of peers (iteration 2). We show estimates of a linear probability model fitted to decisions to violate (0) or to follow (1) the rule, fitted to all data (*cf.* Fig. 3b of the main text). Each treatment enters the regression as a dummy; numbers in the table show shifts in rule-conformity relative to the no-peers baseline treatment as estimated by the respective treatment dummy (with 95% confidence intervals in brackets). We observe an asymmetric effect of peer rule-following and rule-breaking. When all peers follow the rule (and violators are absent; top row) the increase in rule-following is small and insignificant. When all peers violate (and rule-following peers are absent; left column), rule-following decreases substantially and significantly. When peers comprise a mix of rule-following and rule-breaking behaviour, rule-following tends to decrease. The control variables further show that people are consistent in their rule-following (rule-following in iteration 1 positively predicts rule-following in iteration 2); the presence of control questions decreases rule-following; and males tend to be slightly less rule-following than females. The gender effect disappears when the model includes all control variables (*cf.* Table S3). Models considering peer rule-following as a continuous proportion is presented in Table S6. Note that Figure 3b shows the change in rule-following rates in the 27 treatments relative to the no-peer baseline treatment. The data from the no-peers baseline includes a pilot session with 200 participants in which we always included control questions. As a result, the relative frequencies of observations with and without control questions differed across the baseline treatment and the other treatments (see Supplementary Fig. S1 for a full breakdown). The covariate 'control questions' in the statistical analyses (here and in Tables S5,6) accounts for the effect that control questions reduced rule-following (*cf.* Figs. 1c,e). For a fair presentation, Fig. 3b shows averages weighted by proportion of cases including control questions rather than raw averages. P-values for experimental conditions are Bonferroni-corrected.

**Dependent variable**				**Rule conformity (0=no, 1=yes) in iteration 2**			
	**Number of rule-following peers**						
**Number of violating peers**	**0**	**1**	**2**	**3**	**4**	**5**	**6**
**0**	Baseline	0.024<br>(-0.038, 0.087)<br>p=1	0.030<br>(-0.033, 0.092)<br>p=1	0.046<br>(-0.017, 0.109)<br>p=1	0.059<br>(-0.004, 0.122)<br>p=1	0.055<br>(-0.008, 0.117)<br>p=1	0.037<br>(-0.026, 0.100)<br>p=1
**1**	-0.144<br>(-0.207, -0.082)<br>p<0.001	-0.074<br>(-0.138, -0.011)<br>p = 0.674	-0.078<br>(-0.140, -0.015)<br>p=0.478	-0.081<br>(-0.144, -0.017)<br>p=0.407	-0.056<br>(-0.118, 0.007)<br>p=1	-0.030<br>(-0.093, 0.033)<br>p=1	
**2**	-0.193<br>(-0.255, -0.131)<br>p<0.001	-0.136<br>(-0.199, -0.073)<br>p<0.001	-0.094<br>(-0.156, -0.031)<br>p=0.112	-0.126<br>(-0.189, -0.063)<br>p=0.003	-0.077<br>(-0.139, -0.014)<br>0.506		
**3**	-0.208<br>(-0.271, -0.145)<br>p<0.001	-0.147<br>(-0.210, -0.084)<br>p<0.001	-0.170<br>(-0.233, -0.107)<br>p<0.001	-0.144<br>(-0.206, -0.081)<br>p<0.001			
**4**	-0.196<br>(-0.260, -0.132)<br>p<0.001	-0.160<br>(-0.223, -0.098)<br>p<0.001	-0.170<br>(-0.233, -0.107)<br>p<0.001				
**5**	-0.207<br>(-0.270, -0.144)<br>p<0.001	-0.137<br>(-0.200, -0.074)<br>p<0.001					
**6**	-0.221<br>(-0.284, -0.159)<br>p<0.001						
Constant (no peers baseline)	0.518 (0.465, 0.571), p<0.001						
Controls							
Age	0.0002 (-0.001, 0.001), p=0.621						
Male	-0.037 (-0.055, -0.019), p<0.001						
Followed in iteration 1	0.429 (0.410, 0.449), p<0.001						
Control questions (0=no, 1=yes)	-0.071 (-0.091, -0.052), p<0.001						
Observations	7,732						
R^2; Adjusted R^2; Residual Std. Error	0.239; 0.236; 0.410 (df = 7,700)						
F Statistic	77.995 (df = 31; 7,700), p<0.001						

Table S6 | Rule-conformity in the presence of peers (modelling peer rule-conformity rates in iteration 2 as a continuum). We show estimates of linear probability models fitted to decisions to violate (0) or follow (1) the stated rule. The model is similar to the one presented in Table S5, but rather than modelling each treatment as a separate 'level' via a treatment dummy, we here consider the peer behaviour in the different treatments as (continuously varying) proportions of rule-following. The fraction of rule-following peers is calculated as the number of peers following the rule – moving after the traffic light turns green – divided by the total number of peers. The independent variables 'All peers followed' and 'All peers violated' refer to dummies indicating treatments in which all peers either violated or followed the rule to capture any additional effect of observing homogenous rule-following / violation. Note that the number of observations in these analyses is smaller than that in Table S5 because we omit the no-peers baseline, for which the value of 'fraction of rule-following peers' (π) is undefined. The models reveal that the fraction of rule-conforming peers has a positive effect on rule-conformity. Model 1 shows that relative to observing none of the peers following the rule, observing all of them following increases rule-following by 19.2 percentage points. The number of observed peers only matters little. Model 2 shows the strong effects of a single violating peer ('bad apple'). This is evident from the strong positive effect of 'all peers followed'. Moreover, there is no group size effect in the contagiousness of rule violations (the interaction "π × Number of peers" is insignificant). Also, both models show consistency in behaviour across the task iterations: participants' rule-following in iteration 2 of the task is strongly positively associated with rule-following in iteration 1. As before, control questions reduce rule-following. Males are somewhat less likely to follow, but this effect disappears when the model includes all control variables (*cf.* Table S3).

**Dependent variable**	**Rule conformity (0=no, 1=yes) in iteration 2**	
	(1)	(2)
Fraction of rule-following peers (π)	0.192 (0.130, 0.254), p<0.001	0.091 (-0.010, 0.192), p=0.077
Number of peers	-0.013 (-0.022, -0.003), p=0.010	-0.013 (-0.023, -0.002), p=0.016
π × Number of peers	0.009 (-0.006, 0.024), p=0.252	0.016 (-0.0001, 0.032), p=0.052
All peers followed (0 if not, 1 if so)		0.075 (0.033, 0.116), p<0.001
All peers violated (0 if not, 1 if so)		-0.015 (-0.056, 0.026), p=0.471
Constant	0.358 (0.305, 0.412), p<0.001	0.382 (0.313, 0.452), p<0.001
Controls		
Age	0.0003 (-0.001, 0.001), p=0.534	0.0002 (-0.001, 0.001), p=0.575
Male	-0.036 (-0.055, -0.017), p<0.001	-0.036 (-0.055, -0.017), p<0.001
Followed in iteration 1	0.426 (0.406, 0.446), p<0.001	0.426 (0.406, 0.446), p<0.001
Control questions (0=no, 1=yes)	-0.069 (-0.089, -0.049), p<0.001	-0.069 (-0.089, -0.049), p<0.001
Observations	7,312	7,312
R^2	0.231	0.232
Adjusted R^2	0.230	0.232
Residual Std. Error	0.413 (df = 7,305)	0.412 (df = 7,303)
F statistic	313.387 (df = 7; 7,304), p<0.001	245.725 (df = 9; 7,302), p<0.001

Table S7 | Rule-conformity after observing peers (iteration 3). Model setup is identical to Table S6, but now fitted to decisions to follow (1) or violate (0) in the *third* iteration of the task, when participants completed the task alone (see Methods, and Section 1.2 of this SI for a description of the A-B-A design of the task). The model reveals that the effects of observing peers spill over into future behaviour. The effect of observing the behaviour of others ('fraction of following peers', π) is strong (Model 1), albeit slightly less strong than it was in the second iteration, when peers were actually observed (*cf.* Table S6, model 1). Model 2 suggests that this effect is driven by cases where all peers followed the rule. A single violating peer strongly reduces rule-following rates (hence the positive effect of the dummy variable 'all peers followed').

**Dependent variable**	**Rule conformity (0=no, 1=yes) in iteration 3**	
	(1)	(2)
Fraction of following peers in iteration 2 (π)	0.119 (0.060, 0.178), p<0.001	-0.0003 (-0.097, 0.096), p=0.995
Number of peers	0.003 (-0.006, 0.012), p=0.500	0.002 (-0.007, 0.012), p=0.627
π × Number of peers	-0.008 (-0.022, 0.007), p=0.287	0.001 (-0.015, 0.016), p=0.928
All peers followed (0 if not, 1 if so)		0.083 (0.044, 0.123), p<0.001
All peers violated (0 if not, 1 if so)		-0.023 (-0.063, 0.016), p=0.245
Constant	0.504 (0.453, 0.556), p<0.001	0.538 (0.471, 0.604), p<0.001
Controls		
Age	0.001 (-0.0003, 0.001), p=0.219	0.0005 (-0.0003, 0.001), p=0.243
Male	-0.052 (-0.070, -0.033), p<0.001	-0.051 (-0.069, -0.033), p<0.001
Participant followed in iteration 1	0.344 (0.325, 0.363), p<0.001	0.343 (0.324, 0.362), p<0.001
Control questions (0=no, 1=yes)	-0.087 (-0.106, -0.067), p<0.001	-0.087 (-0.106, -0.068), p<0.001
Observations	7,312	7,312
R^2	0.170	0.172
Adjusted R^2	0.169	0.171
Residual Std. Error	0.395 (df = 7,304)	0.394 (df = 7,302)
F statistic	212.993 (df = 7; 7,304), p<0.001	168.076 (df = 9; 7,302), p<0.001

Table S8 | Peer behaviour shifts perceived social appropriateness of rule-breaking and rule-following. This regression complements Extended Data Fig. 3. Numbers show estimates of linear regressions fitted to ratings of violation (model 1) and rule-following (model 2) after observing peers, with 95% confidence intervals in brackets. Treatments differ in the observed peer behaviour (*cf.* Extended Data Fig. 3), and enter the regression as treatment dummies, using the no-peers condition as the baseline. We observe that overall, violations tend to be disapproved of and rule-following tends to be approved. Interestingly, when a group of violators is observed (in particular, the treatment '0 conform, 6 violate'), violation is rated less inappropriate. To a lesser extent, rule-following is approved less when a group of 6 violators is observed. Note that 1 participant was omitted from this analysis as no 'age' was recorded. P-values for treatment comparisons were Bonferroni-corrected.

**Dependent variable**	**Appropriateness ratings**	
	**of rule-breaking**	**of rule-following**
Constant (no-peers baseline)	1.880 (1.593, 2.168), p<0.001	3.557 (3.321, 3.793), p<0.001
0 conform, 6 violate	0.733 (0.499, 0.967), p<0.001	-0.247 (-0.439, -0.055), p=0.013
0 conform, 1 violate	0.336 (0.101, 0.571), p=0.006	-0.041 (-0.234, 0.152), p=0.678
1 conform, 0 violate	-0.066 (-0.295, 0.163), p=0.573	0.069 (-0.119, 0.257), p=0.473
6 conform, 0 violate	-0.062 (-0.291, 0.167), p=0.597	0.077 (-0.111, 0.265), p=0.217
Controls		
Age	-0.009 (-0.015, -0.003), p=0.006	0.004 (-0.001, 0.009), p=0.088
Male	0.088 (-0.059, 0.236), p=0.242	-0.077 (-0.198, 0.045), p=0.217
Observations	490	490
R^2	0.139	0.039
Adjusted R^2	0.129	0.027
Residual Std. Error (df = 483)	0.827	0.680
F Statistic (df = 6; 483)	13.036, p<0.001	3.285, p=0.004

Table S9 | Effects of externalities and punishment on social expectations. We show estimates of linear models fitted to normative evaluations of rule violations (Model 1) and rule-following (Model 2; *cf.* Extended Data Fig. 4a), as well as descriptive beliefs about rule-following (Model 3, estimated rule-following; *cf.* Extended Data Fig. 3b). Across all models, the comparison treatment is the baseline treatment BL). All other treatments do include externalities and vary in the probability that a violation is punished: treatment Externality (EX; 0% probability), treatment Weak Punishment (WP; 10% probability) and treatment Strong Punishment (SP; 90% probability). Externalities are operationalised as participants earning $1.00 for the Red Cross if they follow the rule, and punishment is operationalised as a 'detection mechanism' reducing the participant's earnings in the task to $0 if they violate the rule. The models reveal that overall, appropriateness ratings of violations and rule-following do not vary much with treatment (Models 1 and 2). One exception is that, somewhat surprisingly, violations are less disapproved of in the presence of externalities but no punishment (EX) relative to the no-externalities (BL) baseline. Descriptive expectations show a slight but insignificant increase when externalities are introduced and increase substantially with the introduction of punishment (Model 3).

**Dependent variable**	**(1) Rating of violation**	**(2) Rating of following**	**(3) Descriptive expectations**
Constant (BL)	2.318 (1.937, 2.699) p<0.001	3.238 (2.899, 3.576) p<0.001	39.208 (26.948, 51.469) p<0.001
EX	0.241 (0.008, 0.473) p=0.043	0.049 (-0.158, 0.255) p=0.645	5.058 (-2.150, 12.267) p=0.170
WP	0.148 (-0.085, 0.380) p=0.215	0.019 (-0.187, 0.226) p=0.857	11.468 (4.507, 18.429) p=0.002
SP	0.002 (-0.233, 0.237) p=0.985	0.010 (-0.199, 0.218) p=0.928	14.035 (6.813, 21.258) p<0.001
Controls			
Age	-0.006 (-0.013, 0.001), p=0.112	0.004 (-0.002, 0.011), p=0.197	0.242 (-0.001, 0.485) p=0.052
Male	-0.241 (-0.407, -0.076) p=0.005	0.127 (-0.020, 0.273) p=0.091	4.507 (-0.548, 9.562) p=0.082
Observations	402	402	407
R^2	0.040	0.013	0.060
Adjusted R^2	0.028	0.0003	0.048
Residual	0.843 (df = 396)	0.748 (df = 396)	25.915 (df = 401)
F Statistic	3.289 (df = 5; 396), p=0.006	1.026 (df = 5; 396), p=0.402	5.113 (df = 5; 401), p<0.001

Table S10 | Conditional rule-conformity functions $n(b^n)$ *and* $d(b^d)$ in the presence of externalities and punishment. We show estimates of linear mixed models with 'participant' as random effect, fitted to decisions to break (0) or follow (1) the rule across experimental treatments and conditions shown in Extended Data Figs. 4c,d. Models (1) and (2) are fitted to rule-conformity conditional on normative beliefs, with Model (1) including additional interaction terms testing for slope differences between treatments. Models (3) and (4) are fitted to rule-conformity conditional on descriptive beliefs, with, similarly, Model (3) including additional interaction terms testing for slope differences between treatments. Treatments are the Baseline treatment (BL), Externality (EX), Weak Punishment (WP) and Strong Punishment (SP; see main text for details). 'Condition' reflects the % of people disapproving of violation (for normative beliefs) or the % of people following the stated rule (for descriptive beliefs). These were elicited as quintiles (see Table S4). Fitting logistic rather than linear models lead to very similar results (with the exception that for Model 2, the 95% confidence interval of the main effect of EX no longer brackets zero, but its interaction effect with 'Condition' does). Overall, we observe that introducing externalities and punishment can lead to level shifts in rule-conformity, but conditionality on social expectations remains positive and does not change much between conditions. We observe that in these experiments, males are less likely to conform.

**Dependent variable**	**Conditional rule-conformity (0=no, 1=yes)**			
	**(1) Normative beliefs**	**(2) Normative beliefs**	**(3) Descriptive beliefs**	**(4) Descriptive beliefs**
Constant (BL)	0.466<br>(0.360, 0.572)<br>p<0.001	0.444<br>(0.333, 0.556)<br>p<0.001	0.486<br>(0.372, 0.599)<br>p<0.001	0.452<br>(0.333, 0.571)<br>p<0.001
EX	0.075<br>(-0.003, 0.152)<br>p=0.060	0.148<br>(0.051, 0.246)<br>p=0.003	0.068<br>(-0.012, 0.148)<br>p=0.098	0.126<br>(0.025, 0.226)<br>p=0.014
WP	0.133<br>(0.057, 0.210)<br>p<0.001	0.128<br>(0.032, 0.224)<br>p=0.009	0.092<br>(0.011, 0.172)<br>p=0.027	0.133<br>(0.033, 0.234)<br>p=0.010
SP	0.269<br>(0.192, 0.346)<br>p<0.001	0.287<br>(0.191, 0.384)<br>p<0.001	0.253<br>(0.173, 0.333)<br>p<0.001	0.289<br>(0.189, 0.389)<br>p<0.001
Condition	0.240<br>(0.199, 0.281)<br>p<0.001	0.283<br>(0.201, 0.365)<br>p<0.001	0.172<br>(0.130, 0.214)<br>p<0.001	0.240<br>(0.155, 0.325)<br>p<0.001
EX × Condition		-0.148<br>(-0.264, -0.031)<br>p=0.014		-0.115<br>(-0.235, 0.004)<br>p=0.059
WP × Condition		0.011<br>(-0.105, 0.126)<br>p=0.857		-0.083<br>(-0.203, 0.037)<br>p=0.174
SP × Condition		-0.038<br>(-0.154, 0.078)<br>p=0.523		-0.071<br>(-0.191, 0.048)<br>p=0.241
Controls				
Age	0.001<br>(-0.001, 0.004)<br>p=0.338	0.001<br>(-0.001, 0.004)<br>p=0.338	0.000<br>(-0.003, 0.003)<br>p=0.985	0.000<br>(-0.003, 0.003)<br>p=0.985

Male	-0.169<br>(-0.224, -0.114)<br>p<0.001	-0.169<br>(-0.224, -0.114)<br>p<0.001	-0.100<br>(-0.157, -0.042)<br>p<0.001	-0.100<br>(-0.157, -0.042)<br>p<0.001
Number of participants	606	606	618	618
Observations	3,030	3,030	3,090	3,090
Log Likelihood	-1,440.234	-1,441.873	-1,587.681	-1,591.769
Akaike Inf. Crit.	2,898.469	2,907.746	3,193.362	3,207.537
Bayesian Inf. Crit.	2,952.615	2,979.941	3,247.685	3,279.968

Table S11 | Determinants of rule-conformity in the presence of externalities and punishment. We show estimates of a linear probability model fitted to decisions to violate (0) or follow (1) the stated rule across the four experimental treatments shown in Fig. 4 in the main text. Treatments are Baseline (BL), Externality (EX), Weak Punishment (WP) and Strong Punishment (SP; see main text for details). In the model, the BL treatment is the comparison condition. Our nested treatments sequentially add externalities and punishment. This leads to the prediction of an ordered increase in rule-following rates in the treatments: BL<EX<WP<SP. Two-sided Tukey HSD tests (which is more conservative than a one-sided test) for directional pairwise treatment differences reveal that BL<NP=WP<SP (BL < EX: p=0.026; BL<WP: p=0.015; BL<SP: p<0.001; EX<WP: p=0.998; EX<SP: p<0.001; WP<SP: p<0.001). A logistic model fitted to this data shows the same effects except that the 95% confidence interval of the effect of 'male' brackets zero.

**Dependent variable**		**Rule conformity (0=no, 1=yes)**
BL (comparison)		0.557 (0.493, 0.621), p<0.001
EX		0.066 (0.020, 0.112), p=0.006
WP		0.070 (0.024, 0.116), p=0.003
SP		0.231 (0.184, 0.278), p<0.001
Controls		
	Age	0.000 (-0.001, 0.002), p=0.768
	Male	-0.038 (-0.071, -0.005), p=0.024
Observations		2,003
R^2		0.050
Adjusted R^2		0.048
Residual Std. Error		0.374 (df = 1,997)
F Statistic		21.139 (df = 5; 1,997), p<0.001

4. Experimental instructions shown to participants

Across all screens, we show treatment-specific instructions (e.g., with externalities and punishment) in boxes with grey headers. New screens are indicated with horizontal lines. On-screen buttons are indicated with [square brackets]. Where appropriate, we add comments *** *in italic text between asterisks* ***. These comments were not shown to participants. Note that in the paper we refer to Amazon Mechanical Turk with the abbreviation 'AMT'; in the participant instructions we used the other common shorthand: 'MTurk'.

4.1. Rule-following task (traffic light task) across all experiments

---

Welcome

Thank you for accepting our HIT.

This HIT consists of three Parts. In each Part you can earn points. The number of points you earn depends on your decisions.
Once you have completed this HIT, the computer program will randomly select one of the three Parts to determine your bonus **(20 points = $1.00)**.
That is, only one Part of the HIT will be used to determine your earnings.

You will receive $0.50 on top of however much you earn during the HIT. You will receive a code to enter into MTurk to collect your payment once you have finished.

Note: If you are using a desktop or laptop to complete this HIT, we recommend that you maximize your browser screen before you start.

[continue to instructions]

Instructions Part I

In Part I of this HIT, you control a circle figure that you have to move across the screen over the finish line.

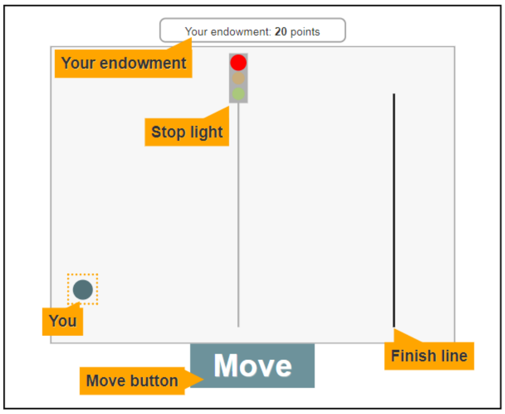

Task screenshot for treatments with externalities

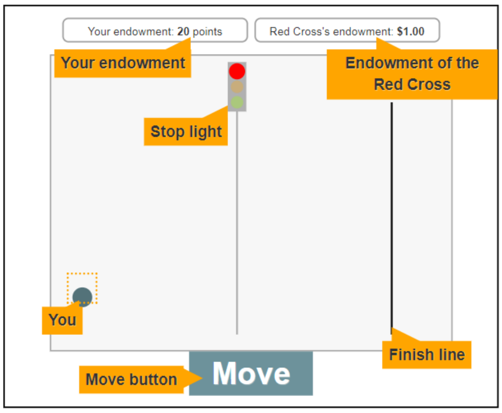

Once you click the **Move** button at the bottom of your screen, your circle will approach the stop light and will stop to wait.
To make your circle move again, again click the **Move** button.

The rule is to wait at the stop light until it turns green.

Your earnings in Part I are determined by the amount of time it takes you to move your circle over the finish line.
Specifically, you begin with an initial endowment of 20 points.

Each second, this endowment will decrease by 1 point until you cross the finish line.

Only shown in treatments with punishment; for the 'Weak Punishment' treatment, only the stated percentage differed (10% instead of 90%)

However, if you do not follow the rule, there is a chance that your earnings will be set to 0.
Specifically, after you have crossed the finish line, there is a **90% chance** that the computer will inspect your actions.
If the computer inspects you and detects that you have not followed the rule, your earnings will be set to 0.

Only shown in treatments with externalities

Finally, in this Part you can also earn money for the **Red Cross**.
The Red Cross has an initial endowment of $1.00.
If you follow the rule, we will pay this $1.00 to the Red Cross.
If you do not follow the rule, the Red Cross's endowment will be reduced to $0.00.

After Part I you will receive instructions for Part II.

Please click below if you read these instructions.
Before the start of the task, a brief quiz will check your understanding of the task.

[Continue]

**** Control questions were only shown for participants in the high saliency condition ****

Control questions

Remember that your earnings in Part I are determined by the amount of time it takes you to move your circle over the finish line.
You begin with 20 points, and each second this endowment will decrease by 1 point until you cross the finish line.

Please answer the following questions.

1. Imagine that you wait to move across the screen until the stop light turns green. You finish the task in 15 seconds.

How many points would you earn? [ ]

Only shown in treatments with externalities
How much money (in $) would the Red Cross earn? [ ]

2. Imagine that you move before the stop light turns green. You finish the task in 4 seconds.

In treatments with punishment the following question was used instead
**2. Imagine that you move before the stop light turns green and you are *not detected*. You finish the task in 4 seconds.**

How many points would you earn? [ ]

Only shown in treatments with externalities
How much money (in $) would the Red Cross earn? [ ]

[Continue]
[Back to instructions]

Ready to start

That is correct! Please click below to proceed to Part I.

[Continue]

**** The following text was shown in an overlay pop-up box which participants could close after 7 seconds. Participants had to click 'OK' before they could start the task (on the same screen) ****

Remember:

- Use the **Move** button to move your circle.
- The rule is to wait at the stop light until it turns green.

[OK]

******

****** In the following screenshots we aim to give an impression of how the task proceeded ******

**** The blue circle starts on the left-hand side of the screen. The endowment is set at 20. This remains the case until the participant clicks 'Move'. This click prompts the circle to move towards the traffic light, and the endowment starts ticking down with 1 point each second. ****

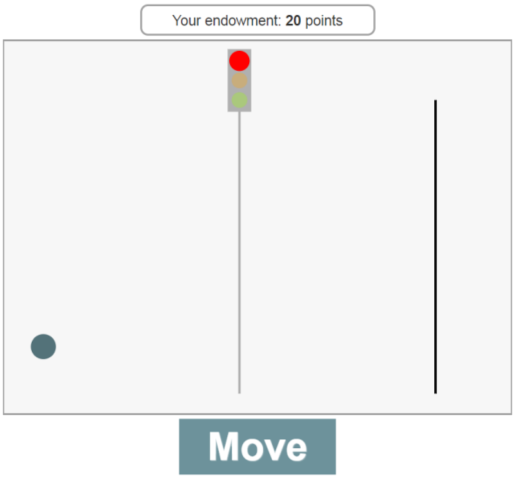

**** After 2 seconds, the endowment has decreased with 2 points and the circle has reached the stop light. The circle has stopped to wait. If the participant clicks 'Move' again, the circle will start moving across the screen across the finish line. ****

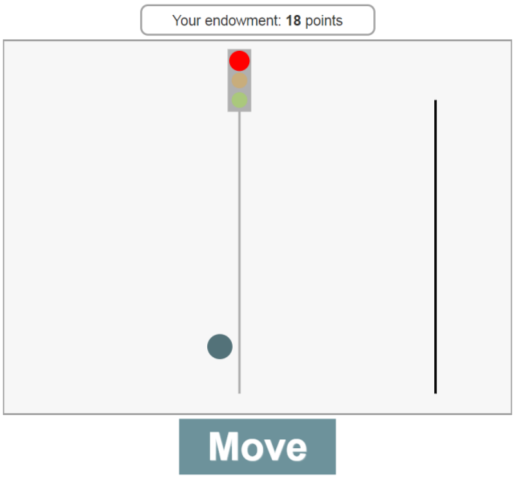

**** After 12 seconds, the endowment has decreased with 12 points (there are 8 points left). The traffic light turns green. ****

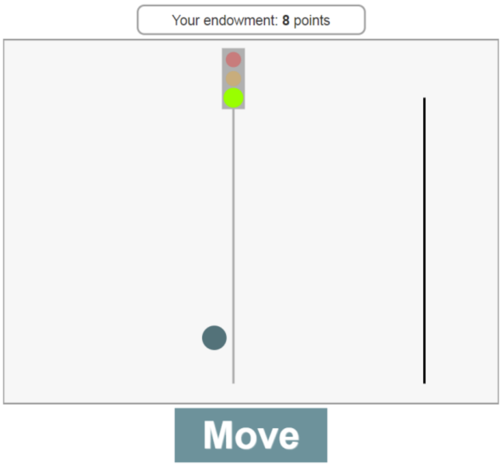

**** The participant has clicked 'Move' again, and the circle moves across the screen and over the finish line. ****

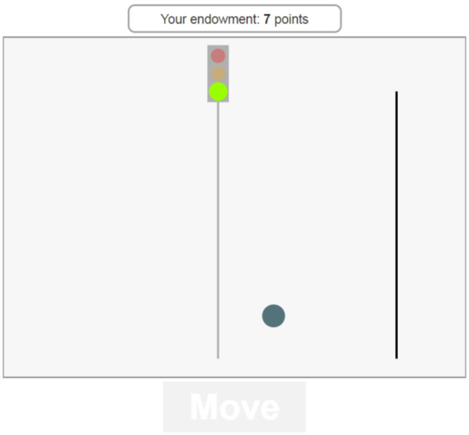

**** After 2 more seconds, the circle has crossed the finish line, and the endowment has stopped decreasing. By clicking 'Submit', the participant moved on to the next screen. ****

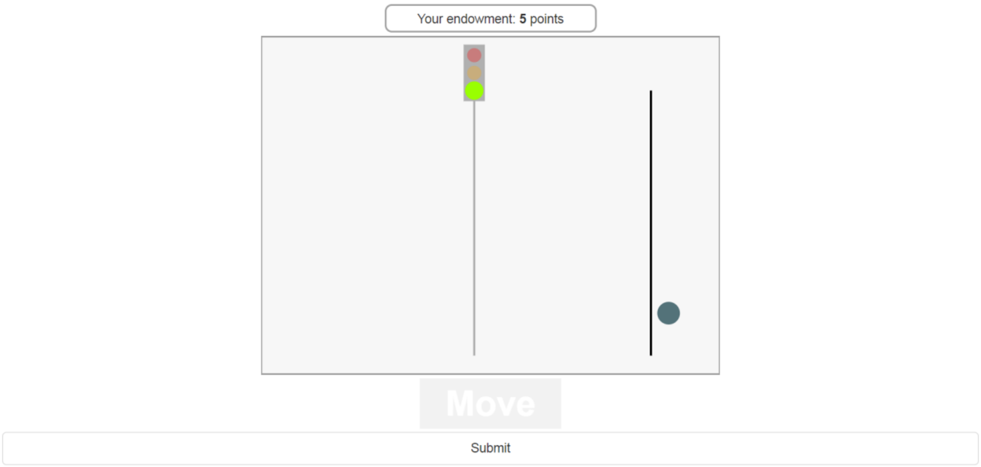

**** For treatments with externalities, the Red Cross's endowment was shown in the same screen. The following two screenshots show examples where the participant respectively violated and conformed with the rule. ****

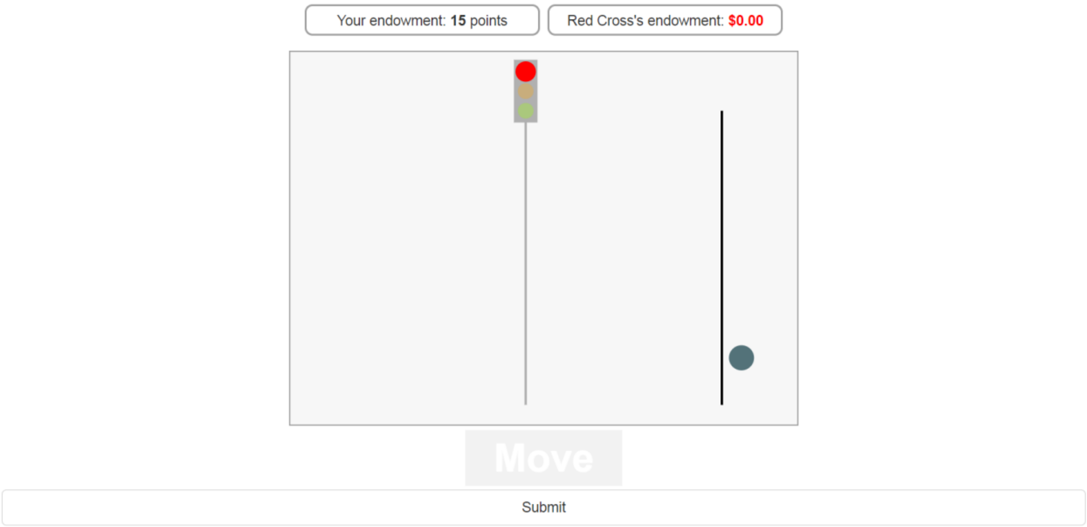

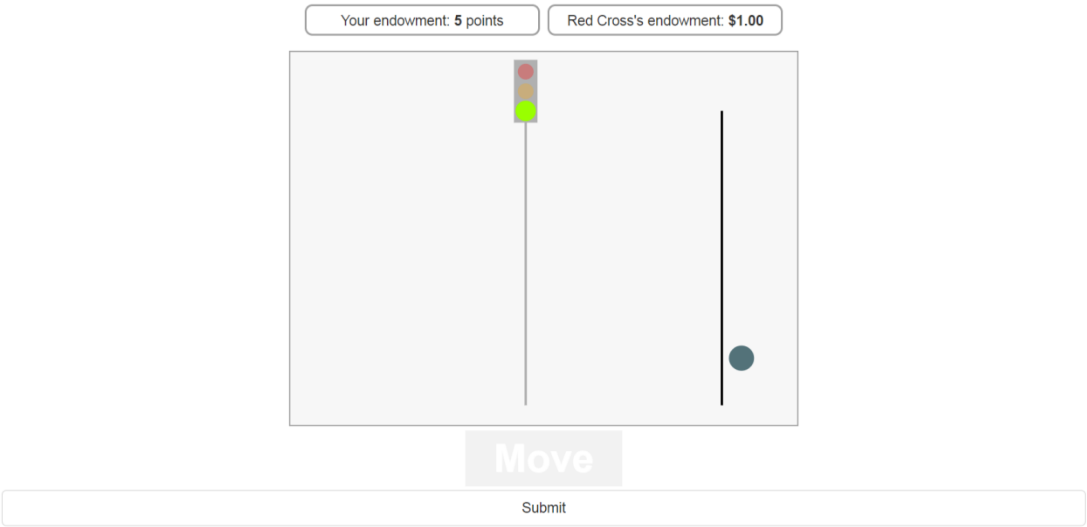

End of Part I

This is the end of Part I.

Only shown in treatments with punishment
You will find out whether the computer has inspected you at the end of the HIT.

Please click below to continue to Part II.

[Continue]

Instructions Part II

Part II of this HIT is similar to Part I. You control a circle figure that you have to move across the screen over the finish line.

Only shown in treatments with peers
In this Part your screen also shows light blue circles. These circles display the movements of other MTurkers.

**** For the treatment without peers, the instruction screenshot for Part II was identical to the screenshot for Part I. Note that the below screenshot also includes externalities; needless to say, these were not shown in the instructions in treatments without externalities. The displayed circles reflected movements of* ***actual*** *MTurkers in previous sessions (who completed the task without peers). We did not use deception in our experiments. ****

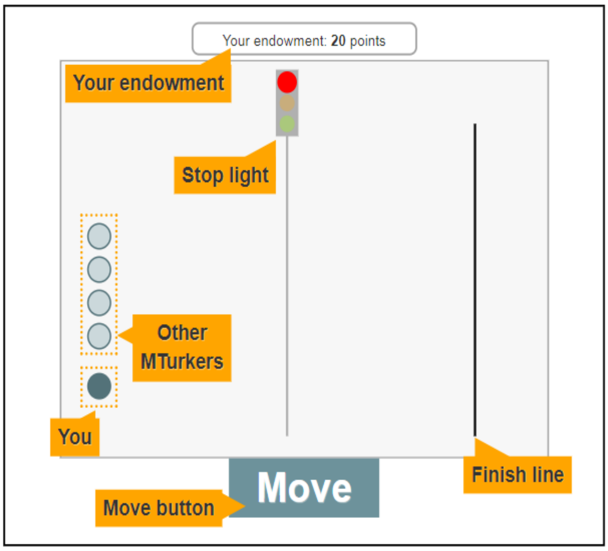

For treatments with externalities, the screenshot also included the Red Cross's endowment

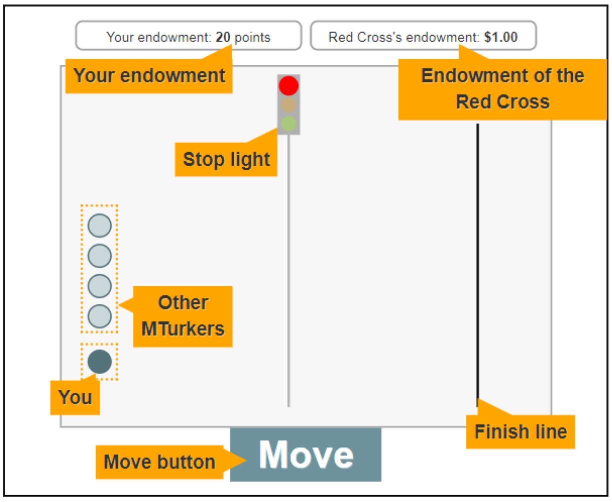

As before, once you click the **Move** button at the bottom of your screen, your circle will approach the stop light and will stop to wait.
To make your circle move again, again click the **Move** button.

The rule is to wait at the stop light until it turns green.

Again, you begin with an initial endowment of 20 points.
Each second, this endowment will decrease by 1 point until you cross the finish line.

Only shown in treatments with punishment; for the 'Weak Punishment' treatment, only the stated percentage differed (10% instead of 90%)
However, if you do not follow the rule, there is a chance that your earnings will be set to 0. Specifically, after you have crossed the finish line, there is a **90% chance** that the computer will inspect your actions. If the computer inspects you and detects that you have not followed the rule, your earnings will be set to 0.

Only shown in treatments with externalities
Finally, in this Part you can also earn money for the **Red Cross**. The Red Cross has an initial endowment of $1.00. If you follow the rule, we will pay this $1.00 to the Red Cross. If you do not follow the rule, the Red Cross's endowment will be reduced to $0.00.

After Part II you will receive instructions for Part III.
Please click below if you are ready to proceed to Part II.

[Continue]

**** The following text was shown in an overlay pop-up box which participants could close after 7 seconds. Participants had to click 'OK' before they could start the task (on the same screen). For conciseness we here show an example in which participants could observe 4 peers, all of whom violated the rule, and moved before the traffic light turned green. Other than displaying the peers, the task proceeded the same as Part I. ****

Remember:

- You control the dark blue circle. The light blue circles display the movements of other MTurkers.
- Use the **Move** button to move your circle.
- The rule is to wait at the stop light until it turns green.

[OK]

**** As in Part I, the circle started on the left-hand side of the screen. As soon as the participant clicked 'Move' their circle as well as the circles of the peers (in this example, 4 peers) started approaching the traffic light. In this example, right after having stopped at the traffic light, each of the 4 peers moved again almost immediately. Once the dark blue circle has moved across the screen and over the finish line, a 'Submit' button will appear. Clicking it will direct the participant to the next screen.****

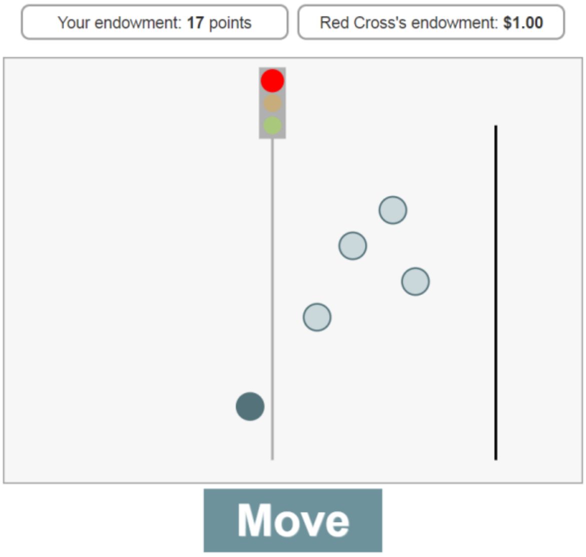

End of Part II

This is the end of Part II.

Only shown in treatments with punishment
You will find out whether the computer has inspected you at the end of the HIT.

Please click below to continue to Part III.

[Continue]

Instructions Part III

Part III of this HIT is similar to Part I and II. You control a circle figure that you have to move across the screen over the finish line.

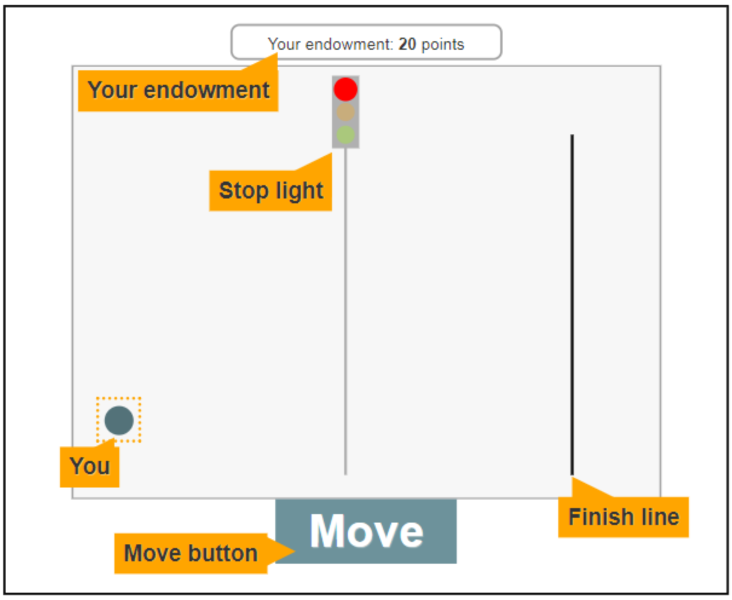

**** NB: In Part III, there were no treatment variations anymore ****

As before, once you click the **Move** button at the bottom of your screen, your circle will approach the stop light and will stop to wait.
To make your circle move again, again click the **Move** button.

The rule is to wait at the stop light until it turns green.

Again, you begin with an initial endowment of 20 points.
Each second, this endowment will decrease by 1 point until you cross the finish line.

Only shown in treatments with punishment; for the ‘Weak Punishment’ treatment, only the stated percentage differed (10% instead of 90%)
However, if you do not follow the rule, there is a chance that your earnings will be set to 0.<br>Specifically, after you have crossed the finish line, there is a **90% chance** that the computer will inspect your actions.<br>If the computer inspects you and detects that you have not followed the rule, your earnings will be set to 0.

Only shown in treatments with externalities
Finally, in this Part you can also earn money for the **Red Cross**.<br>The Red Cross has an initial endowment of $1.00.<br>If you follow the rule, we will pay this $1.00 to the Red Cross.<br>If you do not follow the rule, the Red Cross’s endowment will be reduced to $0.00.

After Part III we ask you to fill out a brief questionnaire.
Please click below if you are ready to proceed to Part III.

[Continue]

**** The following text was shown in an overlay pop-up box which participants could close after 7 seconds. Participants had to click ‘OK’ before they could start the task (on the same screen). The text is identical to the corresponding text in Part I. The same is true for the task. ****

Remember:

- Use the **Move** button to move your circle.
- The rule is to wait at the stop light until it turns green.

[OK]

******

**** Part III is identical to Part I. This example shows again a treatment with externalities. In the screenshot, the participant is 2 seconds into the task. ****

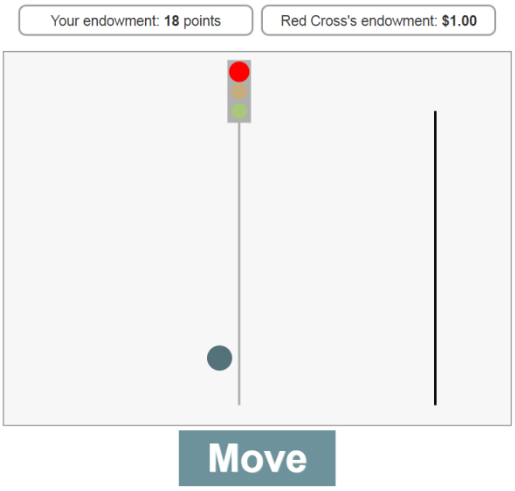

End of Part III

This is the end of Part III.

Only shown in treatments with punishment
You will find out whether the computer has inspected you at the end of the HIT.

Please click below to finish the task.
You can view your earnings after filling out a brief questionnaire.

[Continue]

Questionnaire 1/3

**** For 3031 participants in the behavioural task, the questionnaire was appended to the task itself. For the other 4702 participants, however, this questionnaire was administered in a separate session 1-2 weeks prior to the behavioural task. In the HIT description, we told participants that they should also complete the behavioural task later on to receive payment.* ***

Please answer the following questions.

1. What is your age? [ ]

2. What is your gender?

[Male] [Female]

3. Where did you grow up?

*** dropdown menu including the 50 US States plus the options 'more than one state' and 'outside of the USA' ***

4. How many inhabitants does the village/town/city in which you grew up have?

[Fewer than 1,000] [Between 1,000 and 10,000] [Between 10,000 and 100,000] [between 100,000 and 1,000,000] [More than 1,000,000] [N/A – I grew up in different places]

5. Imagine that you are paired with a randomly chosen other MTurker who also accepted this HIT. Now suppose that each of you would have to make a choice that affects the earnings of both of you. Each of you can choose between two options: **Option A** and **Option B**. The consequences of your choices are as follows:

**If they choose...**	**and you choose...**	**then their earnings are:**	**and your earnings are:**
**Option A**	**Option A**	**$3**	**$3**
**Option A**	**Option B**	**$1**	**$4**
**Option B**	**Option A**	**$4**	**$1**
**Option B**	**Option B**	**$2**	**$2**

You and the other MTurker make this decision only once, and you will not interact in the future.
Now suppose that they make their decision first.

- What would you do if they chose **Option A**?

0 I would choose Option A.
0 I would choose Option B.

- What would you do if they chose **Option B**?

0 I would choose Option A.
0 I would choose Option B.

6. Some people can be described as ‘leaders’ and other people can be described as ‘followers’. Where would you, in general, position yourself on a scale between these descriptions?

A leader 0 0 0 0 0 0 0 A follower

7. Are you generally an impatient person, or someone who always shows great patience?

Very impatient 0 0 0 0 0 0 0 0 0 0 0 Very patient

8. How do you see yourself: are you generally a person who is fully prepared to take risks or do you try to avoid taking risks?

Not at all willing to take risks 0 0 0 0 0 0 0 0 0 0 0 Very willing to take risks

[Continue]

Questionnaire 2/3

*** *The items on this screen represent the Guilt And Shame Proness (GASP) Questionnaire T. R. Cohen, S. T. Wolf, A. T. Panter, C. A. Insko, Introducing the GASP scale: A new measure of guilt and shame proneness. Journal of Personality and Social Psychology 100, 947-966 (2011). doi:10.1037/a0022641* ***

Now you will read about situations that people are likely to encounter in day-to-day life, followed by common reactions to those situations. As you read each scenario, try to imagine yourself in that situation. Then indicate the likelihood that you would react in the say described.

1. After realizing you have received too much change at a store, you decide to keep it because the salesclerk doesn’t notice. What is the likelihood that you would feel uncomfortable about keeping the money?

Very unlikely 0 0 0 0 0 0 0 Very likely

2. You are privately informed that you are the only one in your group that did not make the honor society because you skipped too many days of school. What is the likelihood that this would lead you to become more responsible about attending school?

Very unlikely 0 0 0 0 0 0 0 Very likely

3. You rip an article out of a journal in the library and take it with you. Your teacher discovers what you did and tells the librarian and your entire class. What is the likelihood that this would make you would feel like a bad person?

Very unlikely 0 0 0 0 0 0 0 Very likely

4. After making a big mistake on an important project at work in which people were depending on you, your boss criticizes you in front of your coworkers. What is the likelihood that you would feign sickness and leave work?

Very unlikely 0 0 0 0 0 0 0 Very likely

5. You reveal a friend's secret, though your friend never finds out. What is the likelihood that your failure to keep the secret would lead you to exert extra effort to keep secrets in the future?

Very unlikely 0 0 0 0 0 0 0 Very likely

6. You give a bad presentation at work. Afterwards your boss tells your coworkers it was your fault that your company lost the contract. What is the likelihood that you would feel incompetent?

Very unlikely 0 0 0 0 0 0 0 Very likely

7. A friend tells you that you boast a great deal. What is the likelihood that you would stop spending time with that friend?

Very unlikely 0 0 0 0 0 0 0 Very likely

8. Your home is very messy and unexpected guests knock on your door and invite themselves in. What is the likelihood that you would avoid the guests until they leave?

Very unlikely 0 0 0 0 0 0 0 Very likely

9. You secretly commit a felony. What is the likelihood that you would feel remorse about breaking the law?

Very unlikely 0 0 0 0 0 0 0 Very likely

10. You successfully exaggerate your damages in a lawsuit. Months later, your lies are discovered and you are charged with perjury. What is the likelihood that you would think you are a despicable human being?

Very unlikely 0 0 0 0 0 0 0 Very likely

11. You strongly defend a point of view in a discussion, and though nobody was aware of it, you realize that you were wrong. What is the likelihood that this would make you think more carefully before you speak?

Very unlikely 0 0 0 0 0 0 0 Very likely

12. You take office supplies home for personal use and are caught by your boss. What is the likelihood that this would lead you to quit your job?

Very unlikely 0 0 0 0 0 0 0 Very likely

13. You make a mistake at work and find out a coworker is blamed for the error. Later, your coworker confronts you about your mistake. What is the likelihood that you would feel like a coward?

Very unlikely 0 0 0 0 0 0 0 Very likely

14. At a coworker's housewarming party, you spill red wine on their new cream-colored carpet. You cover the stain with a chair so that nobody notices your mess. What is the likelihood that you would feel that the way you acted was pathetic?

Very unlikely 0 0 0 0 0 0 0 Very likely

15. While discussing a heated subject with friends, you suddenly realize you are shouting though nobody seems to notice. What is the likelihood that you would try to act more considerately toward your friends?

Very unlikely 0 0 0 0 0 0 0 Very likely

16. You lie to people but they never find out about it. What is the likelihood that you would feel terrible about the lies you told?

Very unlikely 0 0 0 0 0 0 0 Very likely

[Continue]

Questionaire 3/3

******* *The items on this screen represent the Ten Item Personality Inventory (TIPI). SD Gosling, PJ Rentfrow, WB Swann Jr. Journal of Research in Personality 37 (6), 2003,504-528****

Here are a number of personality traits that may or may not apply to you. Please indicate for each statement the extent to which *you agree or disagree with that statement*. You should rate the extent to which the pair of traits applies to you, even if one characteristic applies more strongly than the other.

Extraverted, enthusiastic
Disagree strongly 0 0 0 0 0 0 0 Agree strongly

Critical, quarrelsome
Disagree strongly 0 0 0 0 0 0 0 Agree strongly

Dependable, self-disciplined
Disagree strongly 0 0 0 0 0 0 0 Agree strongly

Anxious, easily upset
Disagree strongly 0 0 0 0 0 0 0 Agree strongly

Open to new experiences, complex
Disagree strongly 0 0 0 0 0 0 0 Agree strongly

Reserved, quiet
Disagree strongly 0 0 0 0 0 0 0 Agree strongly

Sympathetic, warm
Disagree strongly 0 0 0 0 0 0 0 Agree strongly

Disorganized, careless
Disagree strongly 0 0 0 0 0 0 0 Agree strongly

Calm, emotionally stable
Disagree strongly 0 0 0 0 0 0 0 Agree strongly

Conventional, uncreative
Disagree strongly 0 0 0 0 0 0 0 Agree strongly

[Continue]

**** Note that we paid out 1 of the 3 Parts at random. ****

Your earnings for this task

The computer has selected Part I to determine your earnings.

In that part, **you waited at the stop light until it turned green.**
You earned 6 points.

These points are worth **$0.30**.

Only shown in treatments with punishment, for participants *who violated the rule*
In that part, **you moved before the stop light turned green.**<br><br>As you know, there was a 90% chance of being detected and have your endowment set to zero.<br>**You were *detected*.** This means that your points are set to 0.<br><br>Your points are worth **$0.00**.

Only shown in treatments with externalities; in this example, the participant *did follow the rule*
In addition, since you followed the rule, **you earned $1.00 for the Red Cross**.<br>Once this study has completed, we will sum the amount that all MTurk participants have earned for the Red Cross and transfer this sum to the Red Cross.<br>Proof of this transfer can be requested from the CeDEx Online Research Team (cedex.nottingham@gmail.com).

Your guaranteed participation fee is: **$0.50**.
So, in total, you have earned **$0.80**.

Note that your participation fee and your bonus will be paid separately.

To receive your earnings, please enter this code into MTurk

1000409

After you have done that, you can close this window.
We thank you for participating in our study.

4.2. Eliciting normative beliefs b^n

Welcome

Thank you for participating in our HIT.
Including the time for reading these instructions, the HIT will take about 8 minutes to complete.

During the HIT, please do not close this window or leave the HIT's web pages in any other way.
If you do close your browser or leave the HIT, you will not be able to re-enter and we will not be able to pay you!

On top of your guaranteed participation fee of $0.50, you can earn a bonus of $2.00 dependent on your responses and the responses of other participants in this HIT.

You will receive a code to collect your payment via MTurk upon completion.

[Continue]

Instructions 1/3

This HIT will ask how socially appropriate certain behaviour is.
By socially appropriate, we mean behaviour that you think most people would agree it is the "correct" thing to do.

Another way to think about what we mean is that if someone were to behave in a socially inappropriate way, then other people might be angry at them.

[Continue]
[Go back]

Instructions 2/3

You will receive a description of a situation in which a person must decide how to act.
This person is another MTurker from the USA taking part in a HIT. We refer to this person as "Person A".
You will be given a description of various possible actions Person A can choose to take.

After you receive the description of the situation, you will be asked to evaluate each of the various possible actions Person A can choose to take.

You must indicate, for each of the possible actions, whether taking that action would be "socially appropriate" or "socially inappropriate".
By socially appropriate, we mean behaviour that you think most other MTurkers from the USA would agree is the "correct" thing to do.

In each of your responses, we would like you to answer as truthfully as possible,
based on your opinions of what constitutes socially appropriate or socially inappropriate behaviour.

[Continue]
[Go back]

Instructions 3/3

Earning a bonus

You will next be given the description of a situation where Person A has to choose how to act.
In this situation, Person A can take two possible actions.
After you read the description of the situation, you must consider each possible action and evaluate how socially appropriate that action is.
So, you will make evaluations for two actions in total.
One of these two actions will be randomly chosen for payment.
This works as follows:

After this HIT has expired we will randomly select one other MTurker
who also participated and evaluated the possible actions of Person A.
The computer will then randomly select one of the two actions.
Your evaluation of this action will be compared with that of the randomly selected MTurker.
(as this HIT is restricted to the USA, the other MTurk participant will also be from the USA).

If your evaluation is the same as theirs, you will receive a bonus of $2.00 for this HIT
(on top of your participation fee of $0.50).
Otherwise, you only receive your participation fee of $0.50.

[Continue]
[Go back]

The situation

Person A is taking part in one of our HITs. This HIT consists of three Parts, in which Person A can earn points.
At the end of the task, these points are converted into dollars (20 points = $1.00).
Person A receives the following instructions for Part I (in italics).

Person A's instructions
In Part I of this HIT, you control a circle figure that you have to move across the screen over the finish line.

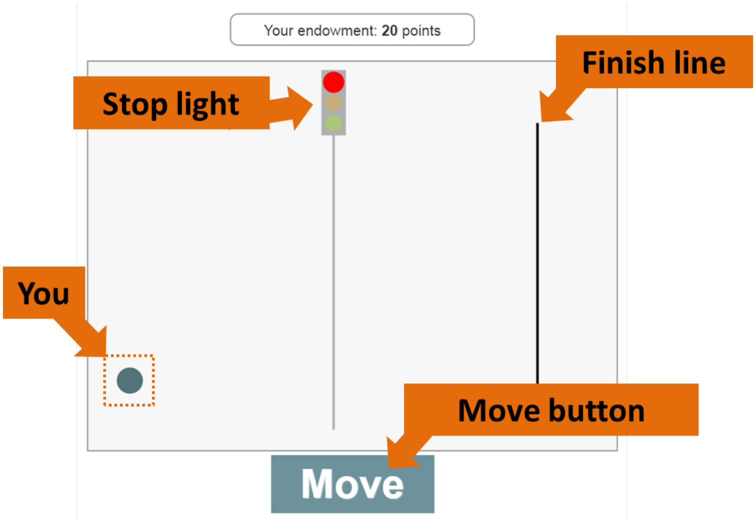

In treatments with externalities, we showed the following task screenshot.

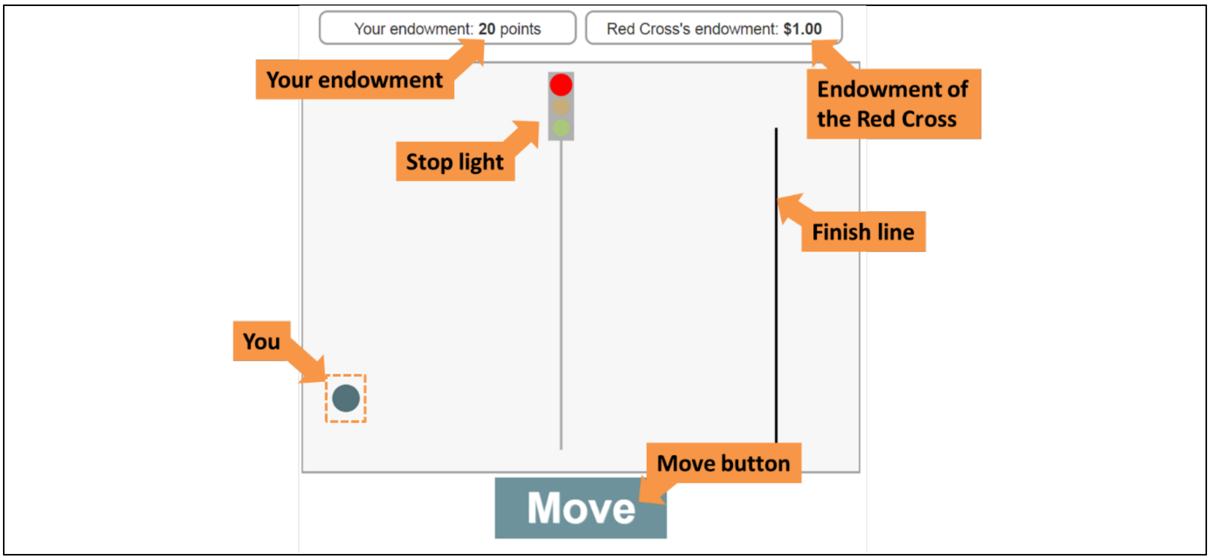

*Once you click the **Move** button at the bottom of your screen, your circle will approach the stop light and will stop to wait.*
*To make your circle move again, again click the **Move** button.*

The rule is to wait at the stop light until it turns green.

Your earnings in Part I are determined by the amount of time it takes you to move your circle over the finish line.
Specifically, you begin with an initial endowment of 20 points.
Each second, this endowment will decrease by 1 point until you cross the finish line.

Only shown in treatments with punishment; for the 'Weak Punishment' treatment, only the stated percentage differed (10% instead of 90%)
However, if you do not follow the rule, there is a chance that your earnings will be set to 0.<br>*Specifically, after you have crossed the finish line, there is a **90% chance** that the computer will inspect your actions.*<br>*If the computer inspects you and detects that you have not followed the rule, your earnings will be set to 0.*

Only shown in treatments with externalities
*Finally, in this Part you can also earn money for the **Red Cross**.*<br>*The Red Cross has an initial endowment of $1.00.*<br>*If you follow the rule, we will pay this $1.00 to the Red Cross.*<br>*If you do not follow the rule, the Red Cross's endowment will be reduced to $0.00.*

[I understand Person A's task. Continue]
[Go back]

Evaluate Person A's possible actions

Right before starting Part I of their task, Person A is reminded of the rule:

The rule is to wait at the stop light until it turns green.

Now imagine Person A is in this situation.
Person A is two seconds into the task - their endowment has decreased from 20 to 18 points.
Their circle has approached the stop light and has stopped to wait.

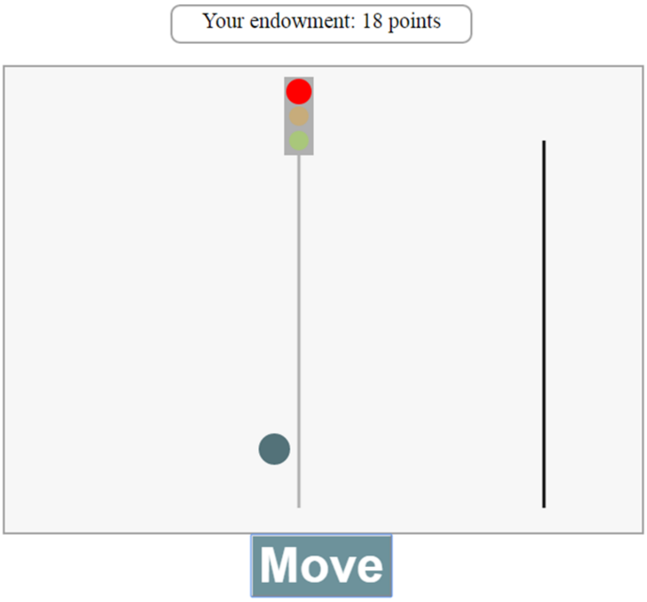

In treatments with externalities, we showed the following task screenshot.

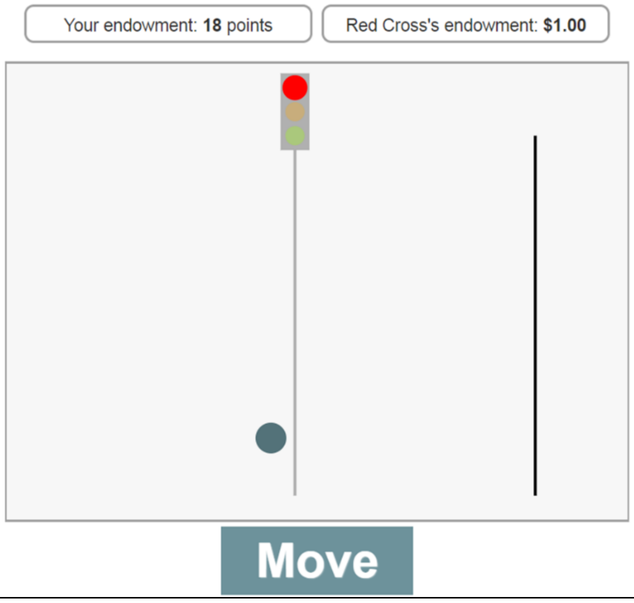

Below you find the actions Person A can take (1. and 2.). Please indicate for each action how socially appropriate you think the action is.

1. Person A moves before the stop light turns green. Person A finishes in 5 seconds and earns 15 points.

In treatments with punishment we used this description instead.
**1. Person A moves before the stop light turns green. Person A finishes in 5 seconds and earns 15 points if they are not detected, and 0 points otherwise.**
In treatments with externalities we *appended* to this to the description.
**The Red Cross's endowment is reduced to $0.00.**

[very socially inappropriate] [somewhat socially inappropriate] [somewhat socially appropriate] [very socially appropriate]

2. Person A waits until stop light turns green. Person A finishes in 14 seconds and earns 6 points.

In treatments with externalities we *appended* to this to the description.
**The Red Cross earns $1.00.**

[very socially inappropriate] [somewhat socially inappropriate] [somewhat socially appropriate] [very socially appropriate]

[Continue]

Questionnaire 1/1

Please fill out this brief questionnaire to finalize this HIT.

What is your gender?

[Male]

[Female]

What is your age?

Person A's instructions you read during your task were taken from a HIT we ran previously on MTurk.
Do you happen to have participated in the HIT that Person A completed,
or participated in a similar HIT in which you had to move your circle across the screen?

[Yes] [No]

[Continue]

HIT completed

This is the end of this HIT.

After this HIT has expired we will randomly select one other MTurker who also participated.
The computer will then randomly select one of the two actions you evaluated.
Your evaluation of this action will be compared with that of the randomly selected MTurker.

If your evaluation is the same as theirs, you will receive a bonus of **$2.00** for this HIT.
Otherwise, you only receive your participation fee of $0.50.

Any bonus you earn will be in addition to your guaranteed participation fee of **$0.50**.

To receive your payment please copy the following code and paste it into MTurk.

1000101

Once you have done that, you can close this window.

Thank you for your participation!

4.3. Eliciting descriptive beliefs b^d

Welcome

Thank you for participating in our HIT.
Including the time for reading these instructions, the HIT will take about 5 minutes to complete.

During the HIT, please do not close this window or leave the HIT's web pages in any other way.
If you do close your browser or leave the HIT, you will not be able to re-enter and we will not be able to pay you.

On top of your guaranteed participation fee of $0.50, you can earn a bonus of $1.00 dependent on your responses.

You will receive a code to collect your payment via MTurk upon completion.

[Continue]

Your task

Your task is to make an estimation about the behaviour of other MTurkers who previously participated in one of our HITs. Specifically, you will be shown the instructions that these other MTurkers received, and we will ask you to estimate how these MTurkers chose to behave.

This HIT will proceed as follows. In the next screen, you will read the instructions that the other MTurkers received, and learn about their choice options. Then, you will make your estimation as to how the other MTurkers chose to behave. Whether or not you will be paid a bonus of $1.00 depends on whether your estimation is accurate. Details will follow later.

[Continue]

Instructions of the previous MTurkers

The previous MTurkers received the following instructions (here shown in italics).

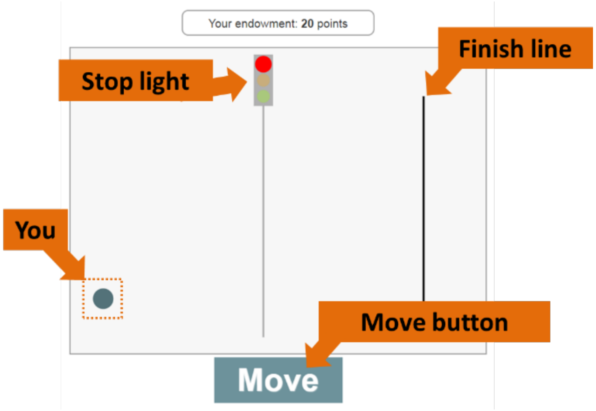

In treatments with externalities, we showed the following task screenshot.

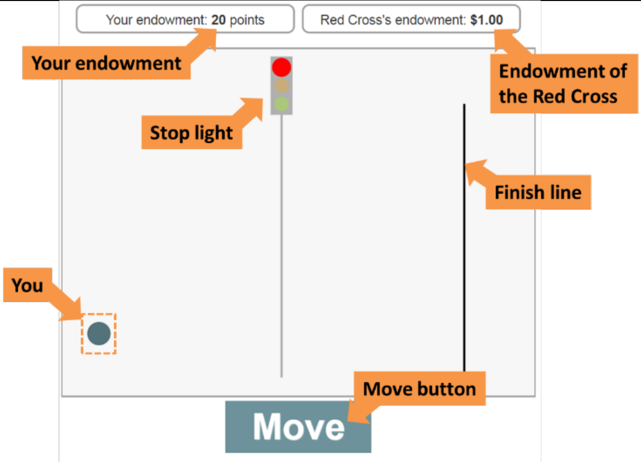

*Once you click the **Move** button at the bottom of your screen, your circle will approach the stop light and will stop to wait.*
*To make your circle move again, again click the **Move** button.*

The rule is to wait at the stop light until it turns green.

Your earnings in Part I are determined by the amount of time it takes you to move your circle over the finish line.
Specifically, you begin with an initial endowment of 20 points.
Each second, this endowment will decrease by 1 point until you cross the finish line.

Only shown in treatments with punishment; for the 'Weak Punishment' treatment, only the stated percentage differed (10% instead of 90%)
However, if you do not follow the rule, there is a chance that your earnings will be set to 0. *Specifically, after you have crossed the finish line, there is a* ***90% chance*** *that the computer will inspect your actions.* *If the computer inspects you and detects that you have not followed the rule, your earnings will be set to 0.*

Only shown in treatments with externalities
Finally, in this Part you can also earn money for the ***Red Cross****.* *The Red Cross has an initial endowment of $1.00.* *If you follow the rule, we will pay this $1.00 to the Red Cross.* *If you do not follow the rule, the Red Cross's endowment will be reduced to $0.00.*

[Click here to confirm that you understand these instructions of the previous MTurkers.]
[Continue]

***** *The Continue button was shown only after clicking the confirmation button above* ***

Instructions of the previous MTurkers

After reading their instructions, the previous MTurkers completed two control questions to ensure that they understood their task (*here shown in italics*).

Remember that your earnings in Part I are determined by the amount of time it takes you to move your circle over the finish line.
You begin with 20 points, and each second this endowment will decrease by 1 point until you cross the finish line.

Please answer the following questions.

1. Imagine that you wait to move across the screen until the stop light turns green. You finish the task in 15 seconds.
How many points would you earn? [correct answer: 5]

Only shown in treatments with externalities
How much money (in $) would the Red Cross earn? [correct answer: 1]

2. Imagine that you move before the stop light turns green. You finish the task in 4 seconds.

In treatments with externalities the following text was used instead
2. *Imagine that you move before the stop light turns* green and you are not detected. *You finish the task in 4 seconds.*

How many points would you earn? [correct answer: 16]

Only shown in treatments with externalities
How much money (in $) would the Red Cross earn? [correct answer: 0]

[Click here to confirm taking note of the control questions completed by the previous MTurkers.]

[Continue]

**** Continue button was shown only after clicking the confirmation button above ****

Make your estimation of the choices of the other MTurkers

Over 500 MTurkers from the USA previously participated in the task. Right before starting, they are reminded of the rule:

The rule is to wait at the stop light until it turns green.

Then they start their task by clicking the Move button once. They start with an initial endowment of 20 points. Each second, this endowment decreases by 1 point. Two seconds into the task their endowment has decreased from 20 to 18 points (note that 20 points = $1.00). Their circle has approached the stop light and has stopped to wait.

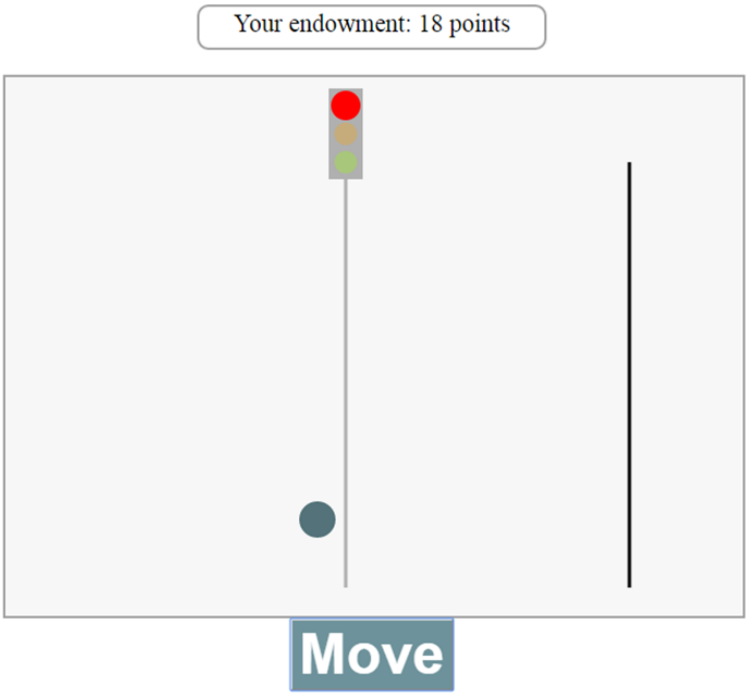

In treatments with externalities, we showed the following task screenshot.

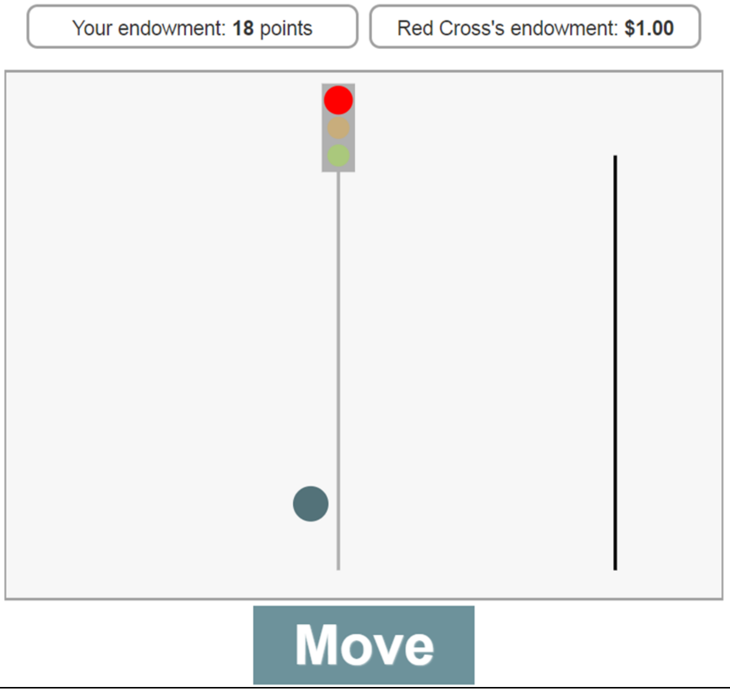

In this situation, the MTurkers could choose either to wait until the stop light turns green, or to move before the traffic light turns green.

Only shown in treatments with punishment; for the 'Weak Punishment' treatment, only the stated percentage differed (10% instead of 90%)
If they move before the traffic light turns green, there is a 90% chance that they are detected and their earnings are set to 0.
Only shown in treatments with externalities; note that the word 'moreover' was only shown in treatments with punishment (this improves the flow of the text).
Moreover, if they move before the traffic light turns green, the Red Cross's endowment is reduced to $0.00.

It is your task now to make an estimation about the choices of the previous MTurkers. If your estimation is accurate, you earn a bonus of $1.00. Specifically, if your estimate is no more than 5 percentage points off, you will receive a bonus. Please make your estimations below.

What percentage of MTurkers waited until the stop light turned green? [ ]

What percentage of MTurkers moved before the stop light turned green? [ ]

[Continue]

****** *The total percentage needed to be 100 for the participant to continue* ***

Your estimation has been recorded. How certain are you of your estimation?

not certain at all 0 0 0 0 0 0 0 completely certain

Questionnaire 1/1

Please fill out this brief questionnaire to finalize this HIT.

What is your gender?

[Male]

[Female]

What is your age?

The instructions you read during your task were taken from a HIT we ran previously on MTurk.
Do you happen to have participated in the HIT these previous MTurkers had completed,
or participated in a similar HIT in which you had to move your circle across the screen?

[Yes] [No]

[Continue]

HIT completed

This is the end of this HIT.

We have recorded your estimation. After this HIT has expired we will reveal the actual percentage of the previous MTurkers waiting for the green light / running the red light. This will be communicated to you in the message which accompanies your bonus payment.

If your estimate was no more than 5 percentage points off the correct answer, you will receive a bonus of $1.00.
If your estimation was more than 5 percentage points off, you will receive a bonus of $0.01.

We kindly ask you to not share or discuss any contents of this HIT nor the bonus message that you will receive (e.g. on AMT forums) because this could compromise our study results.

Your bonus will be paid separately from your guaranteed participation fee of **$0.50**.

To receive your payment please copy the following code and paste it into MTurk.

1000100

Once you have done that, you can close this window.

Thank you for your participation.

4.4. Eliciting the normative conditional rule-conformity function $n(b^n)$

Welcome

Thank you for participating in our HIT.
Including the time for reading these instructions, the HIT will take about 5 minutes to complete.

During the HIT, please do not close this window or leave the HIT's web pages in any other way.
If you do close your browser or leave the HIT, you will not be able to re-enter and we will not be able to pay you.

During this HIT you can earn points. These points will determine your bonus payment for this HIT.
The number of points you earn depends on your decisions.
At the end of the HIT your points will be converted into real money (**20 points are worth $1.00**).

Only shown in treatments with externalities
Your decisions will also determine whether we will make an additional payment of **$1.00 to the Red Cross**. Details will follow later.

In addition to this bonus, you will receive a guaranteed participation fee of **$0.50** upon completion.

You will receive a code to collect your payment via MTurk at the end of this HIT.

[Continue]

Instructions 1/2

In this HIT, you control a circle figure that you have to move across the screen.
Your circle started on the left hand side of the screen. It has approached the stop light and has stopped to wait.
It is your task to move your circle over the finish line (the vertical line on the right hand side of the screen).

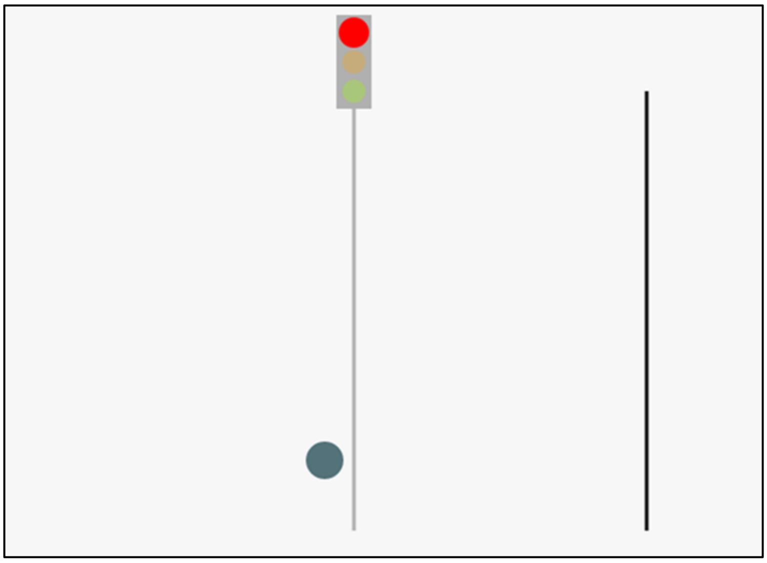

You have to decide when to make your circle move again.
You can choose whether to wait until the stop light turns green or move before the stop light turns green.

The rule is to wait at the stop light until it turns green.

Your earnings are determined by the amount of time it takes you to move your circle over the finish line.

Specifically, if you wait until the stop light turns green, it will take your circle 13 seconds to cross the finish line and your earnings will be 7 points.
If you move before the stop light turns green, it will take your circle 3 seconds to cross the finish line and your earnings will be 17 points.

Only shown in treatments with punishment; for the 'Weak Punishment' treatment, only the stated percentage differed (10% instead of 90%)
However, if you do not follow the rule, there is a chance that your earnings will be set to 0.<br>Specifically, after you have crossed the finish line, there is a **90% chance** that the computer will inspect your actions.<br>If the computer inspects you and detects that you have not followed the rule, your earnings will be set to 0.

Only shown in treatments with externalities
Finally, in this Part you can also earn money for the **Red Cross**.<br>The Red Cross has an initial endowment of $1.00.<br>If you follow the rule, we will pay this $1.00 to the Red Cross.<br>If you do not follow the rule, the Red Cross's endowment will be reduced to $0.00.

[Continue]
[Go back]

Instructions 2/2

Over 100 MTurkers from the USA previously participated in a similar HIT as the one you are participating in today.
These MTurkers were given a description of the task that you have just read in the previous screen.

Only shown in treatments with punishment; for the 'Weak Punishment' treatment, only the stated percentage differed (10% instead of 90%)
They knew that there is a **90% chance** that the computer would inspect one's actions in the task. If the computer inspects one's actions and detects that they have not followed the rule, one's earnings would be set to 0.

Only shown in treatments with externalities
They also knew that, like you, by following or breaking the rule, one could also earn money for the **Red Cross**. By following the rule, one would earn $1.00 for the Red Cross. By breaking the rule, one would earn $0.00 for the Red Cross.

These MTurkers were asked how socially appropriate it is to move before the stop light turns green.

We told these MTurkers that by "socially appropriate" we mean behaviour that most people would agree it is the "correct" thing to do.
Another way to think about what we mean is that if someone were to behave in a socially inappropriate way, then other people might be angry at them.
We have recorded the outcome of this previous HIT.

In today's HIT, we will show you 5 possible outcomes of this previous HIT.
We will ask you, for each outcome, whether you want to **wait** at the stop light or **move** before the stop light turns green.
The 5 possible outcomes differ in the **fraction of previous MTurkers who have indicated that breaking the rule and moving before the stop light turns green is socially *inappropriate***:

A. Between 0% and 20% of previous MTurkers indicated that moving before the stop light turns green is socially *inappropriate*
B. Between 21% and 40% of previous MTurkers indicated that moving before the stop light turns green is socially *inappropriate*
C. Between 41% and 60% of previous MTurkers indicated that moving before the stop light turns green is socially *inappropriate*
D. Between 61% and 80% of previous MTurkers indicated that moving before the stop light turns green is socially *inappropriate*
E. Between 81% and 100% of previous MTurkers indicated that moving before the stop light turns green is socially *inappropriate*

For each possible outcome, you have to choose whether to **wait** at the stop light or **move** before the stop light turns green.

Only one of these 5 choices will be used to compute your bonus earnings.

In treatments with externalities we appended to the previous sentence:
and the payment to the Red Cross

At the end of the HIT we will reveal the actual outcome of the previous HIT (that is, the actual percentage of previous MTurkers who indicated that moving before the stop light turns green is socially inappropriate).
We will use your choice corresponding to this outcome to compute your bonus earnings.

In treatments with externalities we appended to the previous sentence:
and the payment to the Red Cross

For example, suppose that the actual outcome of the previous HIT was that 50% of the previous MTurkers indicated that moving before the stop light turns green is socially inappropriate.
In that case your choice for outcome **C** will be used to compute your bonus earnings.

In treatments with externalities we appended to the previous sentence:
and the payment to the Red Cross

Please click 'Continue' below if you understand these instructions. A brief comprehension check will verify your understanding of your task.

[I understand my task. Continue]
[Go back]

Comprehension check

Please indicate for each of these statements whether they are **correct** or **incorrect**.

Only shown in treatments with punishment; for the 'Weak Punishment' treatment, only the stated percentage differed (10% instead of 90%)
If I do not follow the rule, there is a 90% chance to be detected.<br>[correct] [incorrect]

Only one of the 5 choices I make in this HIT will be used to calculate my bonus earnings.

For treatments with externalities we appended to the end of the sentence:
and the payment to the Red Cross.

[correct] [incorrect]

For that choice, if I wait until the stop light turns green I will earn 7 points.

For treatments with externalities we appended to the end of the sentence:
and the Red Cross will receive $1.00.

[correct] [incorrect]

For that choice, if I move before the stop light turns green I will earn 17 points.

For treatments with punishment we used the following question instead:
For that choice, if I move before the stop light turns green (and I am *not detected*) I will earn 17 points and the Red Cross will receive $0.00.

[correct] [incorrect]

[Continue]
[Go back]

Make your choices

**** The following text was shown in an overlay pop-up box which participants could close down after 7 seconds. Participants had to click 'OK' before they could start the task (on the same screen) ****

Remember:

- The rule is to wait at the stop light until it turns green.

[OK]

******

Choose whether to **wait** at the stop light or **move** before the stop light turns green for each of the 5 possible outcomes listed below:

A. Between 0% and 20% of the previous MTurkers indicated that moving before the stop light turns green is socially *inappropriate*.
[Wait] [Move]

B. Between 21% and 40% of the previous MTurkers indicated that moving before the stop light turns green is socially *inappropriate*.
[Wait] [Move]

C. Between 41% and 60% of the previous MTurkers indicated that moving before the stop light turns green is socially *inappropriate*.
[Wait] [Move]

D. Between 61% and 80% of the previous MTurkers indicated that moving before the stop light turns green is socially *inappropriate*.
[Wait] [Move]

E. Between 81% and 100% of the previous MTurkers indicated that moving before the stop light turns green is socially *inappropriate*.
[Wait] [Move]

[Continue]

Questionnaire 1/1

You have completed the decision making part for this HIT.
Please fill out this brief questionnaire.

What is your gender?

[Male]

[Female]

What is your age? [ ]

The previous MTurkers completed their task earlier this week.

Have you previously participated in a HIT that involved this task or have you participated in a similar HIT in which you had to move your circle across the screen?

[Yes] [No]

[Continue]

HIT completed

This is the end of this HIT.

Of the MTurkers in the previous HIT, **80%** indicated that moving before the stop light turns green is socially inappropriate.

****Note that the percentage was different across treatments to reflect the observed data from the previous experiments ****

This means that we will use your choice **D** to calculate your bonus earnings.
(In D you made your choice for the possible outcome that "between 61% and 80% of previous MTurkers indicated that moving before the stop light turns green is socially inappropriate".)

In that situation, you chose to **move before the stop light turns green**, yielding 17 points.
As you know, 20 points are worth $1.00.

Your bonus earnings are **$0.85**.

In treatments with punishment we showed the following text instead, In this example, the participant violated the rule and was detected; for the 'Weak Punishment' treatment, only the stated percentage differed (10% instead of 90%)
In that situation, you chose to **move before the stop light turns green**, yielding 17 points.<br>As you know, there was a 90% chance of being detected and have your endowment set to zero.<br>**You were *detected*.** This means that your points are set to 0.<br><br>Your points are worth **$0.00**.<br>Your bonus earnings are **$0.00**.

This bonus will be paid on top of your guaranteed participation fee of **$0.50**.

Only shown in treatments with externalities; in this example, the participant *did follow the rule*
Since you did not follow the rule and moved before the stop light turned green, **you did not earn any money for the Red Cross**. Once this study has completed, we will sum the amount that all MTurk participants have earned for the Red Cross and transfer this sum to the Red Cross.<br>Proof of this transfer can be requested from the CeDEx Online Research Team (cedex.nottingham@gmail.com).

To receive your payment please copy the following code and paste it into MTurk.

1000104

Once you have done that, you can close this window. Thank you for your participation.

4.5. Eliciting the descriptive conditional rule-conformity function $d(b^d)$

Welcome

Thank you for participating in our HIT.
Including the time for reading these instructions, the HIT will take about 5 minutes to complete.

During the HIT, please do not close this window or leave the HIT's web pages in any other way.
If you do close your browser or leave the HIT, you will not be able to re-enter and we will not be able to pay you.

During this HIT you can earn points. These points will determine your bonus payment for this HIT.
The number of points you earn depends on your decisions.
At the end of the HIT your points will be converted into real money (**20 points are worth $1.00**).

Only shown in treatments with externalities
Your decisions will also determine whether we will make an additional payment of **$1.00 to the Red Cross**. Details will follow later.

In addition to this bonus, you will receive a guaranteed participation fee of **$0.50** upon completion.
You will receive a code to collect your payment via MTurk at the end of this HIT.

[Continue]

Instructions 1/2

In this HIT, you control a circle figure that you have to move across the screen.
Your circle started on the left hand side of the screen. It has approached the stop light and has stopped to wait.
It is your task to move your circle over the finish line (the vertical line on the right hand side of the screen).

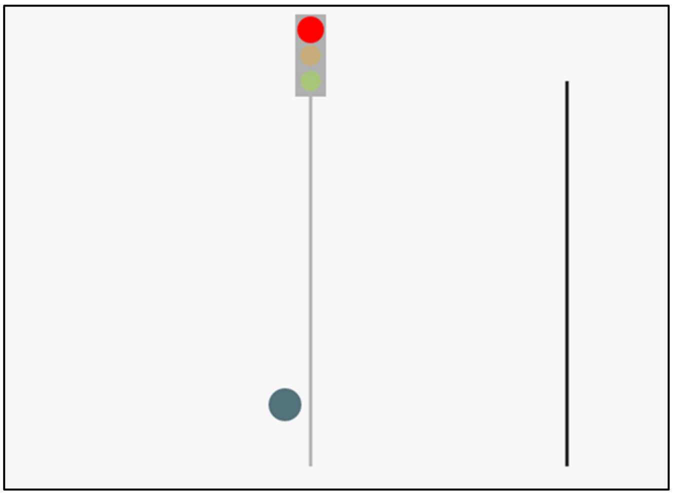

You have to decide when to make your circle move again.
You can choose whether to wait until the stop light turns green or move before the stop light turns green.

The rule is to wait at the stop light until it turns green.

Your earnings are determined by the amount of time it takes you to move your circle over the finish line.

Specifically, if you wait until the stop light turns green, it will take your circle 13 seconds to cross the finish line and your earnings will be 7 points.
If you move before the stop light turns green, it will take your circle 3 seconds to cross the finish line and your earnings will be 17 points.

Only shown in treatments with punishment; for the 'Weak Punishment' treatment, only the stated percentage differed (10% instead of 90%)
However, if you do not follow the rule, there is a chance that your earnings will be set to 0. Specifically, after you have crossed the finish line, there is a **90% chance** that the computer will inspect your actions. If the computer inspects you and detects that you have not followed the rule, your earnings will be set to 0.

Only shown in treatments with externalities
Finally, in this Part you can also earn money for the **Red Cross**. The Red Cross has an initial endowment of $1.00. If you follow the rule, we will pay this $1.00 to the Red Cross. If you do not follow the rule, the Red Cross's endowment will be reduced to $0.00.

[Continue]

[Go back]

Instructions 2/2

Over 500 MTurkers from the USA previously participated in a similar HIT as the one you are participating in today.
These MTurkers also could choose whether to wait until the stop light turns green, or to move before the stop light turns green.

Only shown in treatments with punishment; for the 'Weak Punishment' treatment, only the stated percentage differed (10% instead of 90%)
Also in their case, there was a **90% chance** that the computer would inspect their actions. If the computer inspected them and detected that they had not followed the rule, their earnings would be set to 0.

Only shown in treatments with externalities
Like you, they could also earn money for the **Red Cross**. If they followed the rule, we paid $1.00 to the Red Cross. If they did not follow the rule, the Red Cross's received $0.00. We have recorded the outcome of this previous HIT.

These MTurkers also could choose whether to wait until the stop light turns green, or to move before the stop light turns green.

In today's HIT, we will show you 5 possible outcomes of this previous HIT.
We will ask you, for each outcome, whether you want to **wait** at the stop light or **move** before the stop light turns green.

The 5 possible outcomes differ in the **fraction of previous MTurkers who broke the rule and moved before the stop light turned green**:

A. Between 0% and 20% of previous MTurkers moved before the stop light turned green
B. Between 21% and 40% of previous MTurkers moved before the stop light turned green
C. Between 41% and 60% of previous MTurkers moved before the stop light turned green
D. Between 61% and 80% of previous MTurkers moved before the stop light turned green
E. Between 81% and 100% of previous MTurkers moved before the stop light turned green

For each possible outcome, you have to choose whether to **wait** at the stop light or **move** before the stop light turns green.

Only one of these 5 choices will be used to compute your bonus earnings.

In treatments with externalities, we appended to the previous sentence:
and the payment to the Red Cross

At the end of the HIT we will reveal the actual outcome of the previous HIT (that is, the actual percentage of previous MTurkers who moved before the stop light turned green).
We will use your choice corresponding to this outcome to compute your bonus.

In treatments with externalities, we appended to the previous sentence:
and the payment to the Red Cross

For example, suppose that the actual outcome of the previous HIT was that 50% of previous MTurkers moved before the stop light turned green.
In that case your choice for outcome **C** will be used to compute your bonus earnings.

In treatments with externalities, we appended to the previous sentence:
and the payment to the Red Cross

Please click 'Continue' below if you understand these instructions. A brief comprehension check will verify your understanding of your task.

[I understand my task. Continue]

[Go back]

Comprehension check

Please indicate for each of these statements whether they are **correct** or **incorrect**.

Only shown in treatments with punishment; for the 'Weak Punishment' treatment, only the stated percentage differed (10% instead of 90%)
If I do not follow the rule, there is a 90% chance to be detected.<br>[correct] [incorrect]

Only one of the 5 choices I make in this HIT will be used to calculate my bonus earnings.

For treatments with externalities we appended to the end of the sentence:
and the payment to the Red Cross.

[correct] [incorrect]

For that choice, if I wait until the stop light turns green I will earn 7 points.

For treatments with externalities, we appended to the end of the sentence:
and the Red Cross will receive $1.00.

[correct] [incorrect]

For that choice, if I move before the stop light turns green I will earn 17 points.

For treatments with externalities and punishment, we used the following question instead:
For that choice, if I move before the stop light turns green (and I am *not detected*) I will earn 17 points and the Red Cross will receive $0.00.

[correct] [incorrect]

[Continue]

[Go back]

Make your choices

***** *The following text was shown in an overlay pop-up box which participants could close down after 7 seconds. Participants had to click 'OK' before they could start the task (on the same screen)* *****

Remember:

- The rule is to wait at the stop light until it turns green.

[OK]

******

Choose whether to **wait** at the stop light or **move** before the stop light turns green for each of the 5 possible outcomes listed below:

A. Between 0% and 20% of previous MTurkers moved before the stop light turned green
[Wait] [Move]

B. Between 21% and 40% of previous MTurkers moved before the stop light turned green
[Wait] [Move]

C. Between 41% and 60% of previous MTurkers moved before the stop light turned green
[Wait] [Move]

D. Between 61% and 80% of previous MTurkers moved before the stop light turned green
[Wait] [Move]

E. Between 81% and 100% of previous MTurkers moved before the stop light turned green
[Wait] [Move]

[Continue]

Questionnaire 1/1

You have completed the decision making part for this HIT.
Please fill out this brief questionnaire.

What is your gender?

[Male]

[Female]

What is your age? [ ]

The previous MTurkers completed their task earlier this week.
Have you previously participated in a HIT that involved this task or have you participated in a similar HIT in which you had to move your circle across the screen?

[Yes] [No]

HIT completed

This is the end of this HIT.

Of the MTurkers in the previous HIT, **32%** moved before the stop light turned green.
This means that we will use your choice **B** to calculate your bonus earnings.
(In C you made your choice for the possible outcome that "between 21% and 40% of previous MTurkers moved before the stop light turned green".)

In that situation, you chose to **move before the stop light turns green**, yielding 17 points.
As you know, 20 points are worth $1.00.

Your bonus earnings are **$0.85**.

In treatments with punishment we showed the following text instead, In this example, the participant violated the rule and was detected; for the 'Weak Punishment' treatment, only the stated percentage differed (10% instead of 90%)
In that situation, you chose to **move before the stop light turns green**, yielding 17 points.<br>As you know, there was a 90% chance of being detected and have your endowment set to zero.<br>**You were *detected*.** This means that your points are set to 0.<br><br>Your points are worth **$0.00**.<br>Your bonus earnings are **$0.00**.

This bonus will be paid on top of your guaranteed participation fee of **$0.50**.

Only shown in treatments with externalities; in this example, the participant *did follow the rule*
Since you did not follow the rule and moved before the stop light turned green, **you did not earn any money for the Red Cross**. Once this study has completed, we will sum the amount that all MTurk participants have earned for the Red Cross and transfer this sum to the Red Cross.<br>Proof of this transfer can be requested from the CeDEx Online Research Team (email).

To receive your payment please copy the following code and paste it into MTurk.

1000109

Once you have done that, you can close this window.

Thank you for your participation.

4.6. Eliciting normative expectations before and after observing peer behaviour

---

Welcome

Thank you for participating in our HIT.
Including the time for reading these instructions, the HIT will take about 5 minutes to complete.

During the HIT, please do not close this window or leave the HIT's web pages in any other way.
If you do close your browser or leave the HIT, you will not be able to re-enter and we will not be able to pay you.

On top of your guaranteed participation fee of $0.50, you can earn a bonus of $1.00 dependent on your responses and the responses of other participants in this HIT.

You will receive a code to collect your payment via MTurk upon completion.

[Continue]

Instructions 1/3

This HIT will ask how socially appropriate certain behaviour is.
By socially appropriate, we mean behaviour that you think most people would agree it is the "correct" thing to do.

Another way to think about what we mean is that if someone were to behave in a socially inappropriate way, then other people might be angry at them.

[Continue]

[Go back]

Instructions 2/3

You will receive a description of two situations in which a person must decide how to act.
This person is another MTurker from the USA taking part in a HIT. We refer to this person as "Person A".
You will be given a description of various possible actions Person A can choose to take.

After you receive the description of the situation, you will be asked to evaluate each of the various possible actions Person A can choose to take.

You must indicate, for each of the possible actions, whether taking that action would be "socially appropriate" or "socially inappropriate".
By socially appropriate, we mean behaviour that you think most other MTurkers from the USA would agree is the "correct" thing to do.

In each of your responses, we would like you to answer as truthfully as possible,
based on your opinions of what constitutes socially appropriate or socially inappropriate behaviour.

[Continue]

[Go back]

Instructions 3/3

Earning a bonus

You will next be given the description of two situations where Person A has to choose how to act.
In each situation, Person A can take two possible actions.
After you read the description of each situation, you must consider each possible action and evaluate how socially appropriate that action is.
So, you will make evaluations for four actions in total.
One of these four actions will be randomly chosen for payment.
This works as follows:

After this HIT has expired we will randomly select one other MTurker
who also participated and evaluated the possible actions of Person A.
The computer will then randomly select one of the four actions.
Your evaluation of this action will be compared with that of the randomly selected MTurker.
(as this HIT is restricted to the USA, the other MTurk participant will also be from the USA).

If your evaluation is the same as theirs, you will receive a bonus of $2.00 for this HIT
(on top of your participation fee of $0.50).
Otherwise, you only receive your participation fee of $0.50.

[Continue]

[Go back]

The first situation

Person A is taking part in one of our HITs. This HIT consists of three Parts, in which Person A can earn points.
At the end of the task, these points are converted into dollars (20 points = $1.00).
Person A receives the following instructions for Part I (in italics).

Person A's instructions
In Part I of this HIT, you control a circle figure that you have to move across the screen over the finish line.

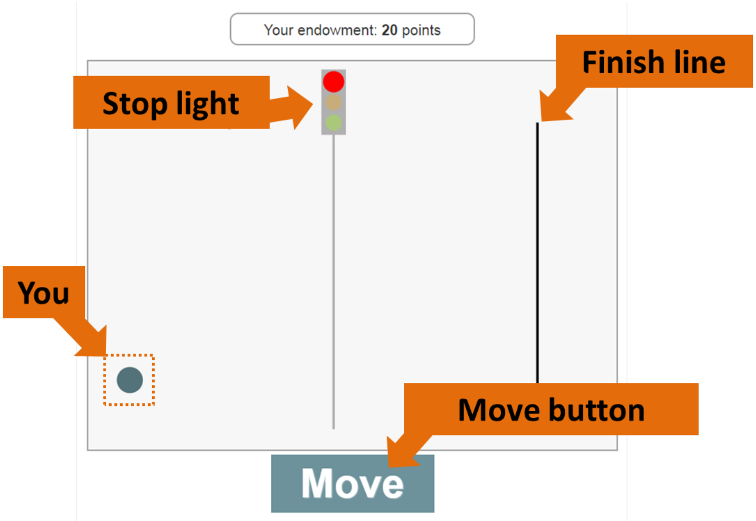

*Once you click the **Move** button at the bottom of your screen, your circle will approach the stop light and will stop to wait.*
*To make your circle move again, again click the **Move** button.*

The rule is to wait at the stop light until it turns green.

Your earnings in Part I are determined by the amount of time it takes you to move your circle over the finish line.
Specifically, you begin with an initial endowment of 20 points.
Each second, this endowment will decrease by 1 point until you cross the finish line.

[I understand Person A's task. Continue]

[Go back]

Evaluate Person A's possible actions

Right before starting Part I of their task, Person A is reminded of the rule:

The rule is to wait at the stop light until it turns green.

Now imagine Person A is in this situation.
Person A is two seconds into the task - their endowment has decreased from 20 to 18 points.
Their circle has approached the stop light and has stopped to wait.

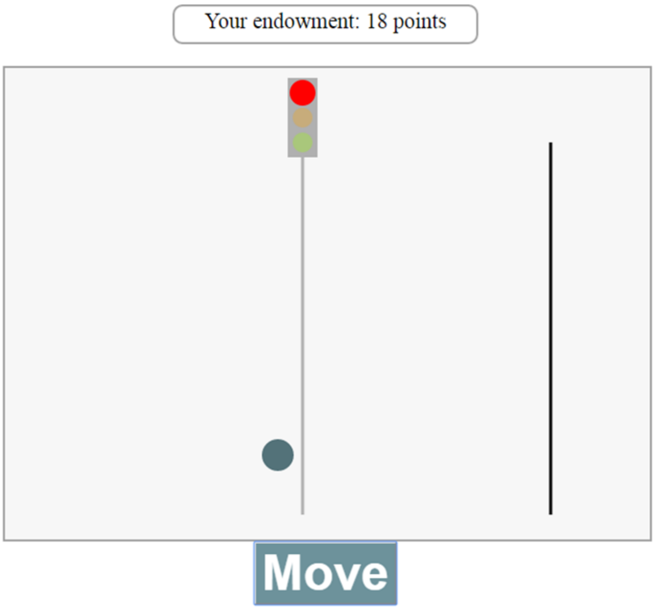

Below you find the actions Person A can take (1. and 2.). Please indicate for each action how socially appropriate you think the action is.

1. **Person A moves before the stop light turns green. Person A finishes in 5 seconds and earns 15 points.**

[very socially inappropriate] [somewhat socially inappropriate] [somewhat socially appropriate] [very socially appropriate]

2. Person A waits until stop light turns green. Person A finishes in 14 seconds and earns 6 points.

[very socially inappropriate] [somewhat socially inappropriate] [somewhat socially appropriate] [very socially appropriate]

[Continue]

The second situation

After completing Part I of their task, Person A starts Part II.
Person A receives the following instructions (in italics).

Person A's instructions

Part II of this HIT is similar to Part I. You control a circle figure that you have to move across the screen over the finish line.

Only shown in treatments with peers
In this Part your screen also shows light blue circles. These circles display the movements of other MTurkers.

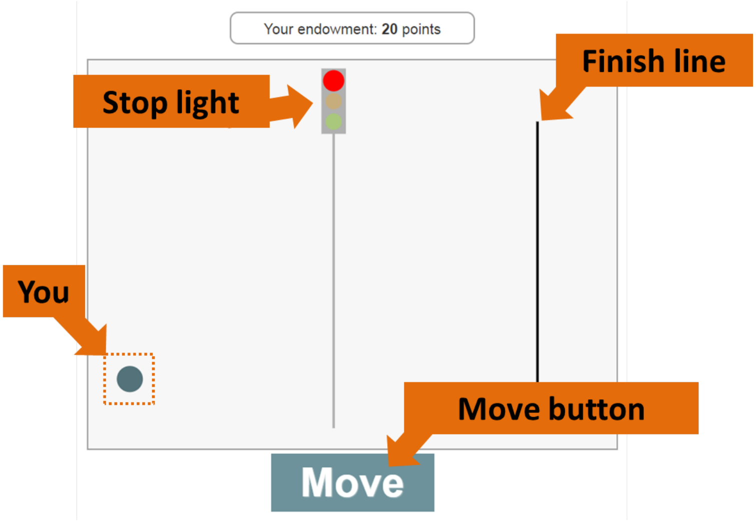

Screenshot shown in treatments with 1 peer

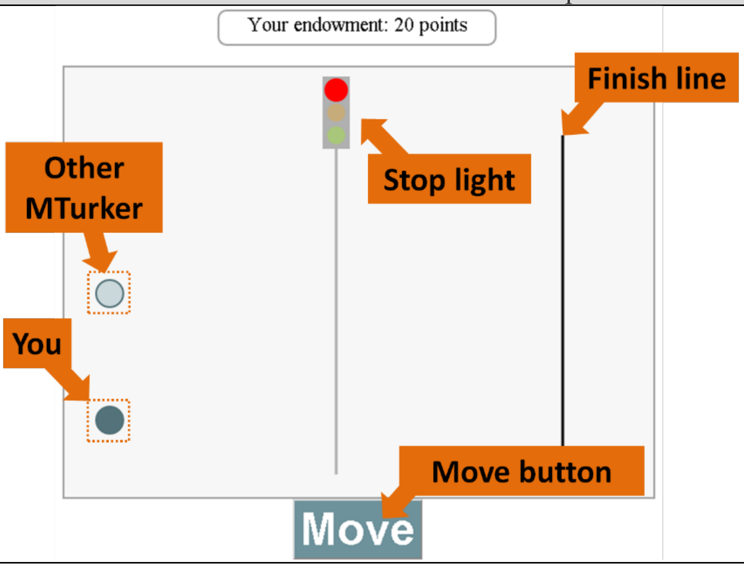

Screenshot shown in treatments with 6 peers

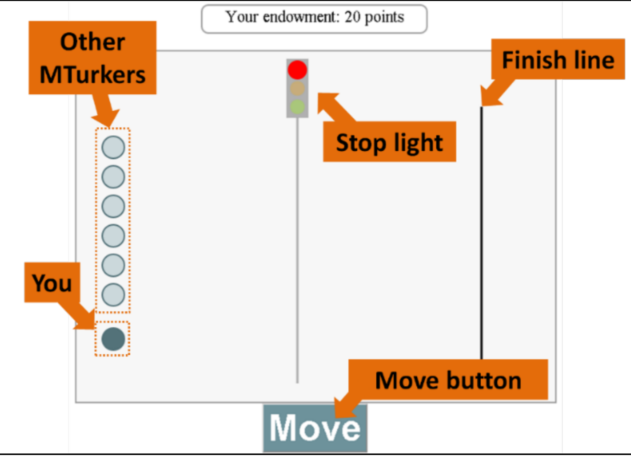

As before, once you click the Move button, your circle will approach the stop light and will stop to wait. To make your circle move again, again click the Move button.

The rule is to wait at the stop light until it turns green.

Again, you begin with an initial endowment of 20 points. Each second, this endowment will decrease by 1 point until you cross the finish line.

[I understand Person A's task. Continue]

Evaluate Person A's possible actions

Right before starting Part II of their task, Person A is reminded of the rule:

The rule is to stop and wait until the stop light turns green.

Now imagine Person A is in this situation.
Person A is two seconds into the task - their endowment has decreased from 20 to 18 points.
Their circle has approached the stop light and has stopped to wait.

Shown in the no-peer (baseline) treatment

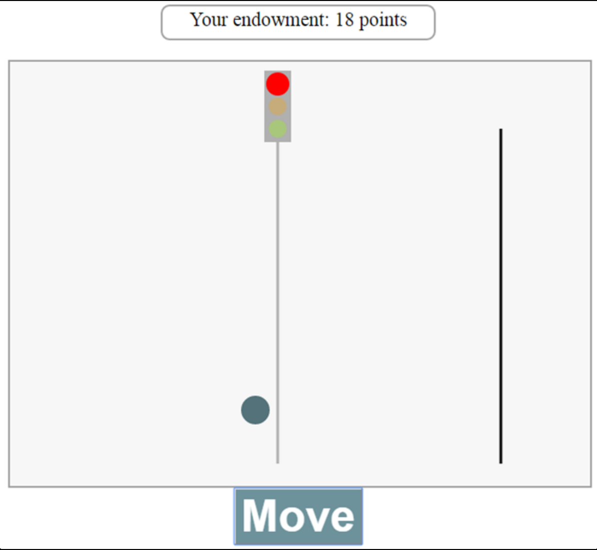

Screenshot shown in the treatment with 6 conforming peers

The circle of the other MTurkers have also stopped to wait.

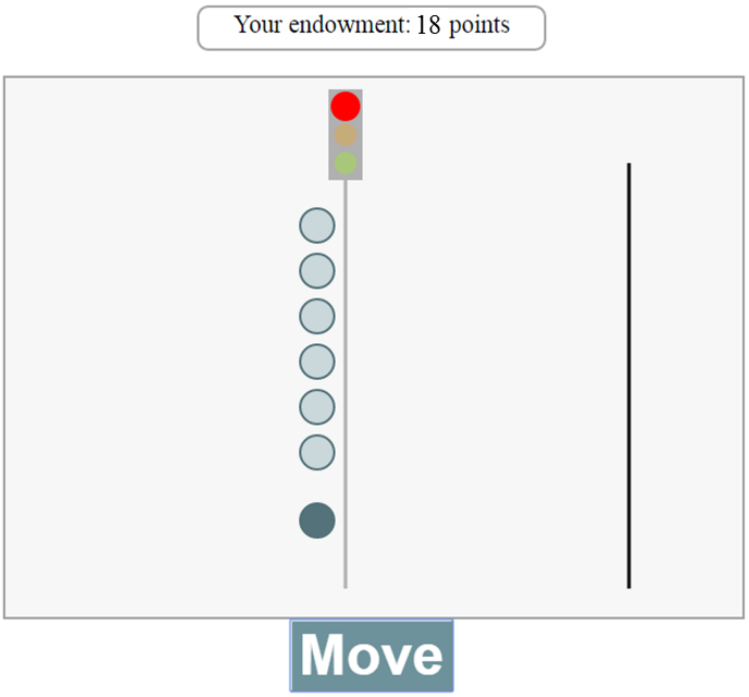

Screenshot shown in the treatment with 1 *conforming* peer

The circle of the other MTurker has also stopped to wait.

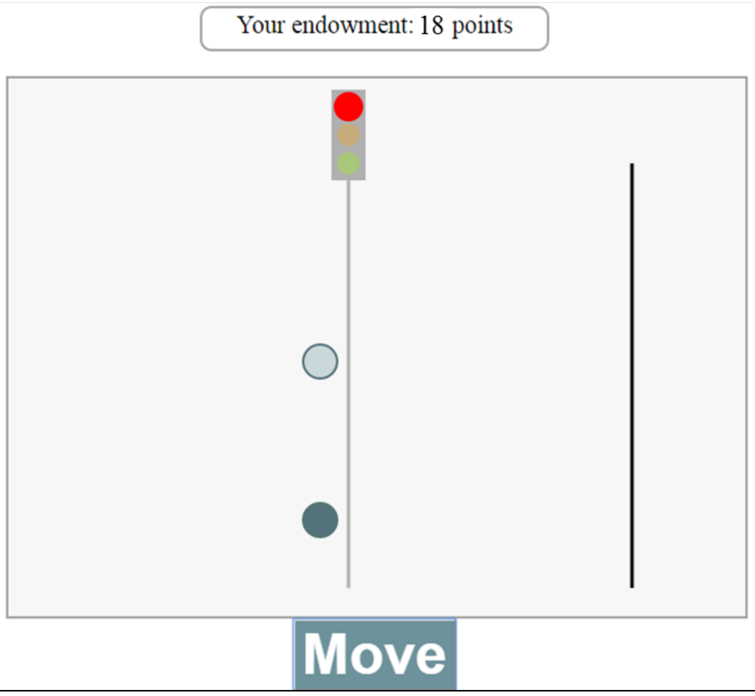

Shown in the treatment with 1 *violating* peer
The circle of the other MTurker has moved before the stop light turned green.

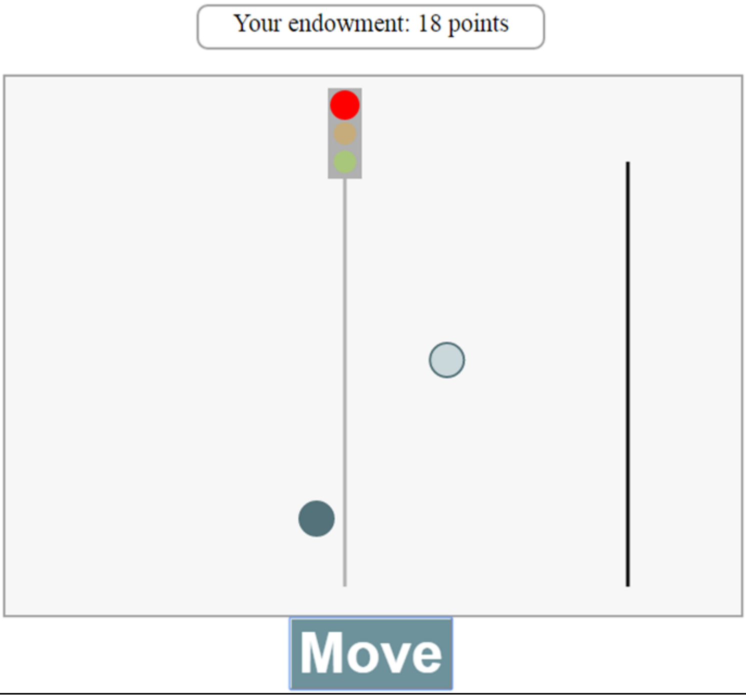

Screenshot shown in the treatment with 6 violating peers
The circles of the other MTurkers have moved before the stop light turned green.

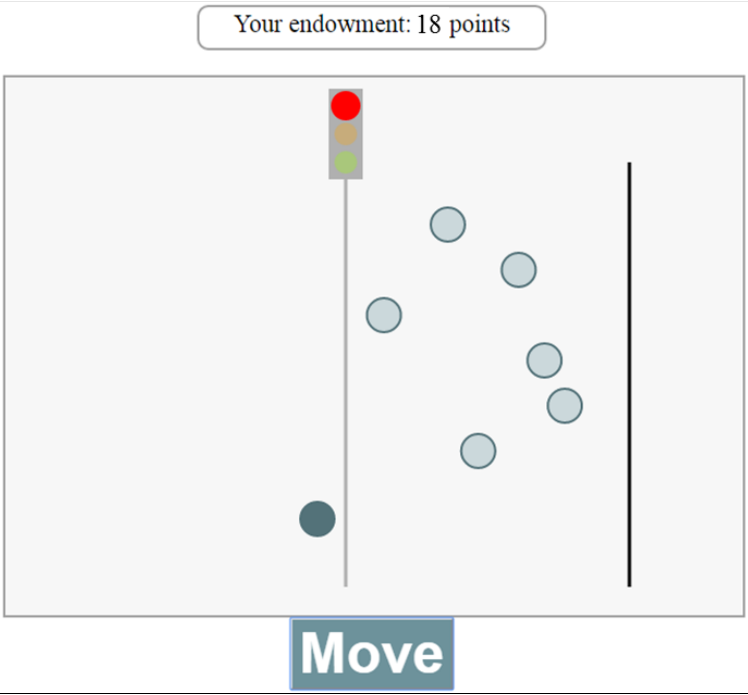

Below you find the actions Person A can take (1. and 2.). Please indicate for each action how socially appropriate you think the action is.

1. Person A moves before the stop light turns green. Person A finishes in 5 seconds and earns 15 points.
[very socially inappropriate] [somewhat socially inappropriate] [somewhat socially appropriate] [very socially appropriate]

2. Person A waits until stop light turns green. Person A finishes in 14 seconds and earns 6 points.
[very socially inappropriate] [somewhat socially inappropriate] [somewhat socially appropriate] [very socially appropriate]

[Continue]

Questionnaire 1/1

Please fill out this brief questionnaire to finalize this HIT.

What is your gender?

[Male]

[Female]

What is your age? [ ]

Person A's instructions you read during your task were taken from a HIT we ran previously on MTurk.
Do you happen to have participated in the HIT that Person A completed,
or participated in a similar HIT in which you had to move your circle across the screen?

[Yes] [No]

[Continue]

HIT completed

This is the end of this HIT.

After this HIT has expired we will randomly select one other MTurker who also participated.
The computer will then randomly select one of the two actions you evaluated.
Your evaluation of this action will be compared with that of the randomly selected MTurker.

If your evaluation is the same as theirs, you will receive a bonus of **$2.00** for this HIT.
Otherwise, you only receive your participation fee of $0.50.

Any bonus you earn will be in addition to your guaranteed participation fee of **$0.50**.

To receive your payment please copy the following code and paste it into MTurk.

1000101

Once you have done that, you can close this window. Thank you for your participation!

4.7. Abstract version of the rule-following task

**** this task is identical to the stop light task (same setup, same instructions, etc). The only differences between the tasks are outlines below ****

Instructions Part I

In Part I of this HIT, you control a circle figure that you have to move across the screen into the black area.

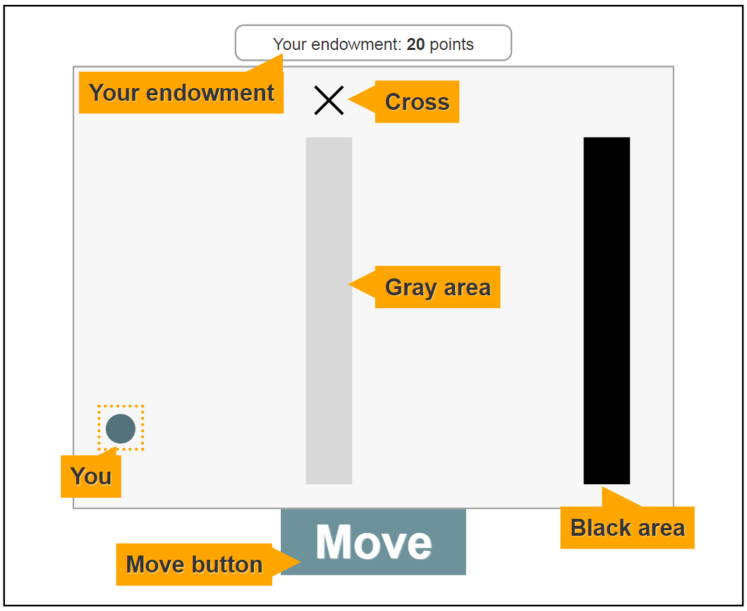

Once you click the **Move** button at the bottom of your screen, your circle will approach the grey area marked with the cross and will stop to wait.
To make your circle move again, again click the **Move** button.

The rule is to wait in the grey area until the cross disappears.

Your earnings in Part I are determined by the amount of time it takes you to move your circle into the black area.
Specifically, you begin with an initial endowment of 20 points.
Each second, this endowment will decrease by 1 point until you reach the black area.

After Part I you will receive instructions for Part II.

Please click below if you read these instructions.
Before the start of the task, a brief quiz will check your understanding of the task.

[Continue]

Control questions

Remember that your earnings in Part I are determined by the amount of time it takes you to move your circle into the black area.
You begin with 20 points, and each second this endowment will decrease by 1 point until you reach the black area.

Please answer the following questions.

1. Imagine that you wait to move across the screen until the cross disappears. You finish the task in 15 seconds.

How many points would you earn?

2. Imagine that you move before the cross disappears. You finish the task in 4 seconds.

How many points would you earn?

[Continue]

[Go back]

**** The task proceeded exactly as the stop light task. Below we show a screenshot of the task start screen. Note that in this abstract version of the task, the cross disappeared after 8 seconds (while in the stop light task, the light turns green after 12 seconds). This change stems from the fact that this abstract task is part of a separate set of experiments. With these separate experiments we examine possible asymmetries in behaviour when rule following manifests as 'omission' (NOT moving before the cross disappears, as in the set of experiments reported here) or when it manifests as 'commission' (moving as soon as the cross disappears). For the latter case, we devised a 'mirrored' version of the task, in which the participant's endowment starts at 0 and increases by 1 every second. The stated rule was to 'move as soon as the cross disappears' (and participants could earn more by violating this rule and wait until the endowment has increased to 20; rule following thus required commission rather than omission). The results of these experiments are beyond the aims and scope of the current paper and will be reported elsewhere. ****

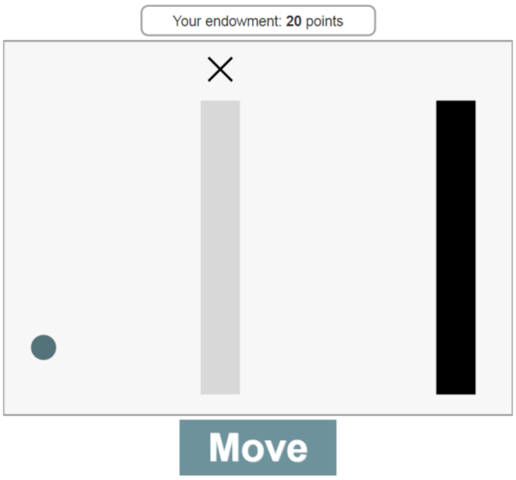

5. Supplementary References

1. Arechar, A. A., Gächter, S. & Molleman, L. Conducting interactive experiments online. *Experimental Economics* **21**, 99-131 (2018).
2. Snowberg, E. & Yariv, L. Testing the waters: Behavior across participant pools. *American Economic Review.* **111**, 687-719 (2021).
3. Giamattei, M., Yahosseini, K. S., Gächter, S. & Molleman, L. Lioness lab: A free web-based platform for conducting interactive experiments online. *J Econ Sci Assoc* **6**, 95-111 (2020).
4. Kimbrough, E. O. & Vostroknutov, A. Norms make preferences social. *Journal of the European Economic Association* **14**, 608-638 (2016).
5. Chesney, T., Chuah, S. H. & Hoffmann, R. Virtual world experimentation: An exploratory study. *Journal of Economic Behavior & Organization* **72**, 618-635 (2009).
6. Prissé, B. & Jorrat, D. Lab vs online experiments: No differences. *Journal of Behavioral and Experimental Economics* **100**, 101910 (2022).
7. Kimbrough, E. O. & Vostroknutov, A. A portable method of eliciting respect for social norms. *Economics Letters* **168**, 147-150 (2018).
8. Gächter, S., Kölle, F. & Quercia, S. Preferences and perceptions in provision and maintenance public goods. *Games and Economic Behavior* **135**, 338-355 (2022).
9. Gächter, S., Starmer, C. & Tufano, F. Measuring the closeness of relationships: A comprehensive evaluation of the 'inclusion of the other in the self' scale. *PLoS ONE* **10**, e0129478 (2015).
10. Arechar, A. A. & Rand, D. G. Turking in the time of covid. *Behav Res* **53**, 2591-2595 (2021).
11. Chmielewski, M. & Kucker, S. C. An mturk crisis? Shifts in data quality and the impact on study results. *Social Psychological and Personality Science* **11**, 464-473 (2019).
12. Hauser, D. J. et al. Evaluating cloudresearch's approved group as a solution for problematic data quality on mturk. *Behav Res* **55**, 3953-3964 (2023).
13. Roth, Y. & Yakobi, O. Attention! Do we really need attention checks? *Journal of Behavioral Decision Making* **37** (2024).
14. Douglas, B. D., Ewell, P. J. & Brauer, M. Data quality in online human-subjects research: Comparisons between mturk, prolific, cloudresearch, qualtrics, and sona. *PLOS ONE* **18**, e0279720 (2023).
15. Stagnaro, M. N. et al. Representativeness versus attentiveness: A comparison across nine online survey samples. *PsyArXiv* (2024).
16. Krupka, E. L. & Weber, R. A. Identifying social norms using coordination games: Why does dictator game sharing vary? *Journal of the European Economic Association* **11**, 495-524 (2013).
17. Fischbacher, U., Gächter, S. & Fehr, E. Are people conditionally cooperative? Evidence from a public goods experiment. *Economics Letters* **71**, 397-404 (2001).
18. Fischbacher, U. & Gächter, S. Social preferences, beliefs, and the dynamics of free riding in public good experiments. *American Economic Review.* **100**, 541–556 (2010).
19. Desmet, P. T. M. & Engel, C. People are conditional rule followers. *Journal of Economic Psychology* **85**, 102384 (2021).
20. Engel, C. How little does it take to trigger a peer effect? An experiment on crime as conditional rule violation. *Journal of Research in Crime and Delinquency* **60**, 455-492 (2023).
21. McBride, M. & Ridinger, G. Beliefs also make social-norm preferences social. *Journal of Economic Behavior & Organization* **191**, 765-784 (2021).
22. Schielzeth, H. et al. Robustness of linear mixed-effects models to violations of distributional assumptions. *Methods in Ecology and Evolution* **11**, 1141-1152 (2020).
23. Team, R. C., *R: A language and environment for statistical computing*. (R Foundation for Statistical Computing https://www.R-project.org/. Vienna, 2020).
24. Bates, D., Mächler, M., Bolker, B. & Walker, S. Fitting linear mixed-effects models using lme4. *arXiv preprint arXiv:1406.5823* (2014).
25. Hothorn, T., Bretz, F. & Westfall, P. Simultaneous inference in general parametric models. *Biometrical Journal* **50**, 346-363 (2008).
26. Vischer, T. et al. Validating an ultra-short survey measure of patience. *Economics Letters* **120**, 142-145 (2013).
27. Gosling, S. D., Rentfrow, P. J. & Swann, W. B. A very brief measure of the big-five personality domains. *Journal of Research in Personality* **37**, 504-528 (2003).
28. Cohen, T. R., Wolf, S. T., Panter, A. T. & Insko, C. A. Introducing the gasp scale: A new measure of guilt and shame proneness. *Journal of Personality and Social Psychology* **100**, 947-966 (2011).
