# Supplementary material for: Why people follow rules
Source: Nat Hum Behav. 2025 May 26;9(7):1342–54. doi: 10.1038/s41562-025-02196-4 (PMC12283409; doi:10.1038/s41562-025-02196-4)
Supplement: Supplementary file 2 — Reporting Summary [file 41562_2025_2196_MOESM2_ESM.pdf]

nature portfolio

Corresponding author(s): Simon Gaechter

Last updated by author(s): 8 March 2025

Reporting Summary

Nature Portfolio wishes to improve the reproducibility of the work that we publish. This form provides structure for consistency and transparency in reporting. For further information on Nature Portfolio policies, see our Editorial Policies and the Editorial Policy Checklist.

Statistics

For all statistical analyses, confirm that the following items are present in the figure legend, table legend, main text, or Methods section.

n/a	Confirmed	
☐	☒	The exact sample size (*n*) for each experimental group/condition, given as a discrete number and unit of measurement
☐	☒	A statement on whether measurements were taken from distinct samples or whether the same sample was measured repeatedly
☐	☒	The statistical test(s) used AND whether they are one- or two-sided<br>*Only common tests should be described solely by name; describe more complex techniques in the Methods section.*
☐	☒	A description of all covariates tested
☐	☒	A description of any assumptions or corrections, such as tests of normality and adjustment for multiple comparisons
☐	☒	A full description of the statistical parameters including central tendency (e.g. means) or other basic estimates (e.g. regression coefficient) AND variation (e.g. standard deviation) or associated estimates of uncertainty (e.g. confidence intervals)
☐	☒	For null hypothesis testing, the test statistic (e.g. F, t, r) with confidence intervals, effect sizes, degrees of freedom and P value noted<br>*Give P values as exact values whenever suitable.*
☒	☐	For Bayesian analysis, information on the choice of priors and Markov chain Monte Carlo settings
☒	☐	For hierarchical and complex designs, identification of the appropriate level for tests and full reporting of outcomes
☒	☐	Estimates of effect sizes (e.g. Cohen's *d*, Pearson's *r*), indicating how they were calculated

Our web collection on statistics for biologists contains articles on many of the points above.

Software and code

Policy information about availability of computer code

Data collection	We used custom code of the open source software LIONESS Lab. All experiments are available in editable form via https://lioness-lab.org
Data analysis	Data and analysis code can be found on OSF (https://doi.org/10.17605/OSF.IO/7WZ4F)<br>Data analysis software:<br>R (version 4.2.0); R packages: lme4 (Bates et al 2015) and multcomp (Hothorn et al 2008). Citations are provided in the Supplementary Information.

For manuscripts utilizing custom algorithms or software that are central to the research but not yet described in published literature, software must be made available to editors and reviewers. We strongly encourage code deposition in a community repository (e.g. GitHub). See the Nature Portfolio guidelines for submitting code & software for further information.

Data

Policy information about availability of data

All manuscripts must include a data availability statement. This statement should provide the following information, where applicable:

- Accession codes, unique identifiers, or web links for publicly available datasets
- A description of any restrictions on data availability
- For clinical datasets or third party data, please ensure that the statement adheres to our policy

The data and analysis code are deposited at https://doi.org/10.17605/OSF.IO/7WZ4F

Research involving human participants, their data, or biological material

Policy information about studies with human participants or human data. See also policy information about sex, gender (identity/presentation), and sexual orientation and race, ethnicity and racism.

Reporting on sex and gender	We only asked for gender and 49% identified as male. Tables S1 and S2 in the Supplementary Information contain full details for each of our experiments.
Reporting on race, ethnicity, or other socially relevant groupings	We did not collect any race, ethnicity or other socially relevant grouping information; we only asked for age and gender. Details of distributions of participant age and gender are found in Tables S1 and Table S2 (Supplementary Information).
Population characteristics	A large majority of participants were volunteer Amazon Mechanical Turk US American workers; mean age 34.6 years; 49% male. Details for each experiment are reported in Tables S1 and S2 (Supplementary Information). In Experiment 1 we further show data from 103 UK student participants (mean age: 21.1; 46% male).
Recruitment	Participants were volunteers recruited on Amazon Mechanical Turk. Participation was restricted to US American residents of 18 years and older. Descriptions of the studies were kept vague to avoid any self-selection bias.
Ethics oversight	All studies reported in this paper were approved by the Research Ethics Committee, School of Economics, University of Nottingham (protocol ID 030_ERC_AP_MT).

Note that full information on the approval of the study protocol must also be provided in the manuscript.

Field-specific reporting

Please select the one below that is the best fit for your research. If you are not sure, read the appropriate sections before making your selection.

☐ Life sciences ☒ Behavioural & social sciences ☐ Ecological, evolutionary & environmental sciences

For a reference copy of the document with all sections, see nature.com/documents/nr-reporting-summary-flat.pdf

Behavioural & social sciences study design

All studies must disclose on these points even when the disclosure is negative.

Study description	Data are quantitative data resulting from experiments we conducted using our custom-made software.
Research sample	A large majority of our study participants were US American volunteers recruited on the Amazon Mechanical Turk platform. They earned a flat fee for completing the study, and could earn an additional bonus payment dependent on their decisions (see Methods). We also report on data from 103 UK student participants who took part in the CeDEx lab at the University of Nottingham. They also earned a flat fee upon completion, plus a bonus that depended on their behaviour in the task (for details, see Section 1 of the Supplementary Information).
Sampling strategy	We used convenience sampling on Amazon Mechanical Turk to ensure we had sufficient participants from the same pool for our range of experiments.
Data collection	Data were collected online using a custom code of the software LIONESS Lab. Participants were recruited on the Amazon Mechanical Turk platform. Participants were anonymous and not known to the researcher (for details, see Section 1 of the Supplementary Information). The researcher was not blinded to experimental conditions and/or the study hypotheses.
Timing	Data for Experiments 1 - 3 were collected between 2014 and 2016; Experiments 4 were conducted in 2018.
Data exclusions	No data were excluded if participants completed the study. Incomplete submissions were discarded.
Non-participation	No participants declined participation.
Randomization	For Experiments 1 and 3 participants first completed the rule-following task on their own (with or without control questions; Experiment 1) and were then randomly assigned to one of the 28 treatments, which varied in the behaviour of "peers" (Experiment 3), For each of the tasks in Experiment 2, participants were randomly assigned to a treatment related to either normative or descriptive beliefs. In Experiments 4, participants were randomly assigned to one of the conditions BL, EX, WP, or SP described in the main text.

Reporting for specific materials, systems and methods

We require information from authors about some types of materials, experimental systems and methods used in many studies. Here, indicate whether each material, system or method listed is relevant to your study. If you are not sure if a list item applies to your research, read the appropriate section before selecting a response.

Materials & experimental systems

n/a	Involved in the study
☒	☐ Antibodies
☒	☐ Eukaryotic cell lines
☒	☐ Palaeontology and archaeology
☒	☐ Animals and other organisms
☒	☐ Clinical data
☒	☐ Dual use research of concern
☒	☐ Plants

Methods

n/a	Involved in the study
☒	☐ ChIP-seq
☒	☐ Flow cytometry
☒	☐ MRI-based neuroimaging

Plants

Seed stocks	*Report on the source of all seed stocks or other plant material used. If applicable, state the seed stock centre and catalogue number. If plant specimens were collected from the field, describe the collection location, date and sampling procedures.*
Novel plant genotypes	*Describe the methods by which all novel plant genotypes were produced. This includes those generated by transgenic approaches, gene editing, chemical/radiation-based mutagenesis and hybridization. For transgenic lines, describe the transformation method, the number of independent lines analyzed and the generation upon which experiments were performed. For gene-edited lines, describe the editor used, the endogenous sequence targeted for editing, the targeting guide RNA sequence (if applicable) and how the editor was applied.*
Authentication	*Describe any authentication procedures for each seed stock used or novel genotype generated. Describe any experiments used to assess the effect of a mutation and, where applicable, how potential secondary effects (e.g. second site T-DNA insertions, mosiacism, off-target gene editing) were examined.*
